# Prognostic and predictive value of pre-treatment blood-based inflammatory biomarkers in patients with urothelial carcinoma treated with immune checkpoint inhibitors: a systematic review and meta-analysis

**DOI:** 10.3389/fimmu.2025.1554048

**Published:** 2025-03-17

**Authors:** Ádám Széles, András Kubik, Szilárd Váncsa, Viktor Grünwald, Boris Hadaschik, Nándor Ács, Péter Hegyi, Péter Nyirády, Tibor Szarvas

**Affiliations:** ^1^ Department of Urology, University of Duisburg-Essen and German Cancer Consortium (DKTK)-University Hospital Essen, Essen, Germany; ^2^ Department of Urology, Semmelweis University, Budapest, Hungary; ^3^ Center for Translational Medicine, Semmelweis University, Budapest, Hungary; ^4^ Institute for Translational Medicine, Medical School, University of Pécs, Pécs, Hungary; ^5^ Institute of Pancreatic Diseases, Semmelweis University, Budapest, Hungary; ^6^ Department of Obstetrics and Gynecology, Semmelweis University, Budapest, Hungary

**Keywords:** urothelial carcinoma, bladder cancer, immune checkpoint inhibitor, CRP, NLR, PLR

## Abstract

**Background and objectives:**

The therapeutic landscape of locally advanced or metastatic urothelial carcinoma (mUC) is rapidly evolving, and immune checkpoint inhibitors (ICI) have become an integral part of the standard therapy. However, the majority of patients do not benefit from this treatment. Hence, finding prognostic and predictive biomarkers may improve therapeutic decision-making. The aim of this study was to analyze the prognostic and predictive significance of liquid biomarkers (NLR, CRP, PLR, and LDH) in mUC patients treated with ICI.

**Methods:**

We collected articles from PubMed, Cochrane, and Embase databases with primary outcomes of overall survival (OS), progression-free survival (PFS) and objective response rate (ORR).

**Key findings and limitations:**

We compiled data from a total of 6,673 ICI-treated patients with locally advanced or mUC from 31 articles. Pooled univariate analysis demonstrated that high pre-treatment NLR is significantly associated with worse OS (HR: 2.19; 95% CI: 1.80-2.68) and PFS (HR: 1.90; 95% CI: 1.57-2.31). Similarly, elevated CRP levels were associated with worse OS (HR: 1.75; 95% CI: 1.37-2.24) and PFS (HR: 1.58; 95% CI: 1.26-1.99).

**Conclusions and clinical implications:**

Elevated pre-treatment NLR, CRP, PLR, and LDH are significantly associated with worse OS and PFS in ICI-treated urothelial carcinoma patients, suggesting that they have potential prognostic and predictive value in treatment decisions.

**Patient summary:**

In this systematic review and meta-analysis we summarized the existing data on inflammatory laboratory biomarkers and their potential impact on immunotherapy outcomes in urothelial cancers.

**Systematic Review Registration:**

https://www.crd.york.ac.uk/prospero/, identifier CRD42022291449.

## Introduction

Urothelial carcinoma (UC) is one of the most prevalent human malignancies worldwide (1). Locally advanced or metastatic UC (mUC) is a clinically challenging, highly aggressive disease characterized by short survival rates and limited treatment options. Platinum-based chemotherapy has been the only therapeutic option for mUC for decades (2). However, only ~50% of patients show radiographic response to this chemotherapy, and only 20% of patients will survive longer than two years, while serious side effects of this agent can deeply affect its administration (3).

In 2016 and 2017, two innovative immune checkpoint inhibitor (ICI) therapies, atezolizumab (from the IMvigor 210 study) and pembrolizumab (from the KEYNOTE-045 study), were introduced for patients with UC that had progressed during or after platinum-based chemotherapy (4, 5). These therapies achieved objective response rates of 15-29%, which are substantially higher than the response rates of less than 10% observed with other second-line chemotherapies. Notably, patients who responded to these treatments experienced a durable response lasting longer than 12 months, an unprecedented improvement at this stage of treatment. In 2017, both drugs were also approved for first-line use in platinum-ineligible patients based on the IMvigor 210 and KEYNOTE-052 studies (5, 6). In addition, maintenance therapy with avelumab became available for patients who initially responded to platinum chemotherapy (4). Overall, ICIs represent a promising new therapeutic strategy that offers a lasting therapeutic effect and prolonged survival for a subgroup of patients. Furthermore, other novel targeted therapies have become available, such as the FGFR-inhibitor erdafitinib and the Nectin-4 targeting enfortumab vedotin, both used most recently in third-line treatment, and enfortumab vedotin in combination with pembrolizumab in the first-line mUC treatment (7, 8). Predicting the response to ICI therapy is of significant clinical importance.

Despite the positive results of ICIs in mUC, survival and response rates remain heterogeneous, with less than 30% of mUC patients responding to ICI therapy (4, 5). This problem highlights the need for clinically easy-to-reach reliable biomarkers that can help us design appropriate treatment solutions and sequences. Currently, only a few biomarkers are available to predict ICI therapy. PD-L1 immunohistochemistry is one of the most widely used tissue-based biomarkers currently used to decide between carboplatin and ICI in cisplatin-ineligible mUC patients. However, the negative predictive value of this method is poor, as PD-L1 negative patients may also respond well to ICI therapy. The clinical feasibility of other biomarkers such as microsatellite instability (MSI) and tumor mutational burden (TMB) remains questionable (9, 10). Furthermore, tissue-based biomarkers in general present further challenges due to their difficult and invasive availability and the inability to monitor treatment.

Blood-based biomarkers of inflammation, such as neutrophil-to-lymphocyte ratio (NLR), C-reactive protein (CRP), platelet-to-lymphocyte ratio (PLR), and lactate dehydrogenase (LDH) are routinely available biomarkers that have been widely investigated in different cancers (11–13). In the era of immunotherapy, these biomarkers have also received significant attention, as ICIs can reactivate immune response in the tumor tissue, but can also act as antagonists of systemic inflammation (14, 15). Especially in mUC, a well-known immunogenic malignancy, inflammation plays a critical role in the pathophysiology (16). Therefore, in this study, we aimed to systematically investigate the prognostic relevance of NLR, CRP, PLR, and LDH in ICI-treated locally advanced and mUC patients.

## Methods

The study was conducted according to the Preferred Reporting Items for Systematic Reviews and Meta-analyses (PRISMA) 2020 recommendations (**Supplementary Table 1**) (17), and followed the Cochrane Handbook (18). The study protocol was registered on PROSPERO (Nr. CRD42022291449).

### Literature search and selection of studies

The electronic databases PubMed, EMBASE, and Cochrane Library were screened on February 28, 2023, using the searchkey in **Supplementary Text 1** in **Data Sheet 1**. No filters were used in the search. In addition, references to articles included were screened to identify additional potentially eligible studies.

Two independent authors (AS and KA) performed the systematic selection process. Disagreements were resolved by a third author (SzV). References were screened using Endnote X^9^ (Clarivate Analytics, Philadelphia, PA, USA) and assessed by title, abstract, and full text.

### Eligibility criteria

We used the PECO framework to formulate our research question. We included original English-language studies that examined (P) patients with ICI-treated urothelial carcinoma and (E and C) compared the hazard of high and low serum or plasma NLR, CRP, LDH, and PLR levels for (O) overall survival (OS) or progression-free survival (PFS) and ORR (objective response rate). For the assessed biomarkers, we used cut-off values based on the definitions in the original articles. The following exclusion criteria were used: reviews, comments, letters, meta-analyses, systamatic reviews, animal experiments, and conference abstracts were excluded.

### Data extraction

Data were obtained by reading full-text articles by two independent authors (AS, KA). Parameters extracted were first name of author, publication year, tumor location (upper *vs.* lower urinary tract), type of ICI therapy, country of sample/data collection, type of study, cohort size, patient age, sex, ECOG performance status, cut-off values for NLR, CRP, LDH and PLR, follow-up time, OS, PFS, and ORR. For eligible studies, the article provided calculated hazard ratios (HR) with 95% confidence intervals (CI). In addition, objective response rates (ORR) were also evaluated when available.

### Quality assessment and evaluation of evidence

Risk of bias was assessed by two independent authors using the Quality in Prognostic Studies (QUIPS) tool (19). The study attrition domain was assessed only for prospective studies. The RobVisR tool was used to summarize the results of the evaluations (**Supplementary Table 3**) (20). GRADEpro™ program was used to evaluate the evidence (**Supplementary Table 4**) (21).

### Synthesis methods

1. All statistical analyses were performed with R (R Core Team 2023, v4.3.2), using the meta (22) package for basic meta-analysis calculations and plots, and dmetar (23) package for additional influental analysis calculations and plots.
2. For time-to-event data, hazard ratio (HR) was used for the effect size measure with 95% confidence interval (CI). To calculate the pooled HR, we calculated the logarithm of HR and its SE from the available data following the methodology of Tierney et al. (24).
3. We extracted or calculated the total number of patients and events (“raw data”) from available studies. Using these data, we calculated odds ratios (ORs) with 95% confidence intervals (CIs) as the effect size measure. Results are reported as the odds of the event in the experimental group compared to the control group.
4. Pooled OR based on raw data was calculated using the Mantel-Haenszel method (25, 26). The pooled HR was calculated using the inverse variance weighting method (on a logarithmic scale).
5. We used a Hartung-Knapp adjustment (27) for CIs (28). To estimate the heterogeneity variance measure (τ2), for raw data OR calculation, we used the Paule-Mandel method (29) (recommended by Veroniki et al. (30)) with the Q-profile method for the confidence interval. For HRs, the restricted maximum-likelihood estimator was used with the Q profile method for the confidence interval (23) (30).
6. Results were considered statistically significant if the pooled CI did not contain the null value. We summarized the findings in forest plots. Where applicable, and where the number of studies was sufficiently large and not too heterogeneous, we also reported the prediction intervals (i.e., the expected range of effects of future studies) of results. In addition, between-study heterogeneity was described by the Higgins & Thompson’s statistics (31).
7. We conducted subgroup analyses by line of therapy (first-line, second-line *vs.* mixed), drug (atezolizumab, pembrolizumab and mixed), study design (prospective *vs.* retrospective), and study site (singlecenter *vs.*multicenter). For subgroup analysis, we used a fixed-effects “plural” model (aka. mixed-effects model). We assumed that all subgroups had a common τ2 value as we did not anticipate differences in the between-study heterogeneity between the subgroups, and the number of studies was relatively small in some subgroups (recommended by Borenstein et al. (32)., The “Cochrane Q” test (an omnibus test) was used to assess differences between subgroups (23). The null hypothesis was rejected at the 5% significance level.

## Results

### Search and selection

**Figure 1:**
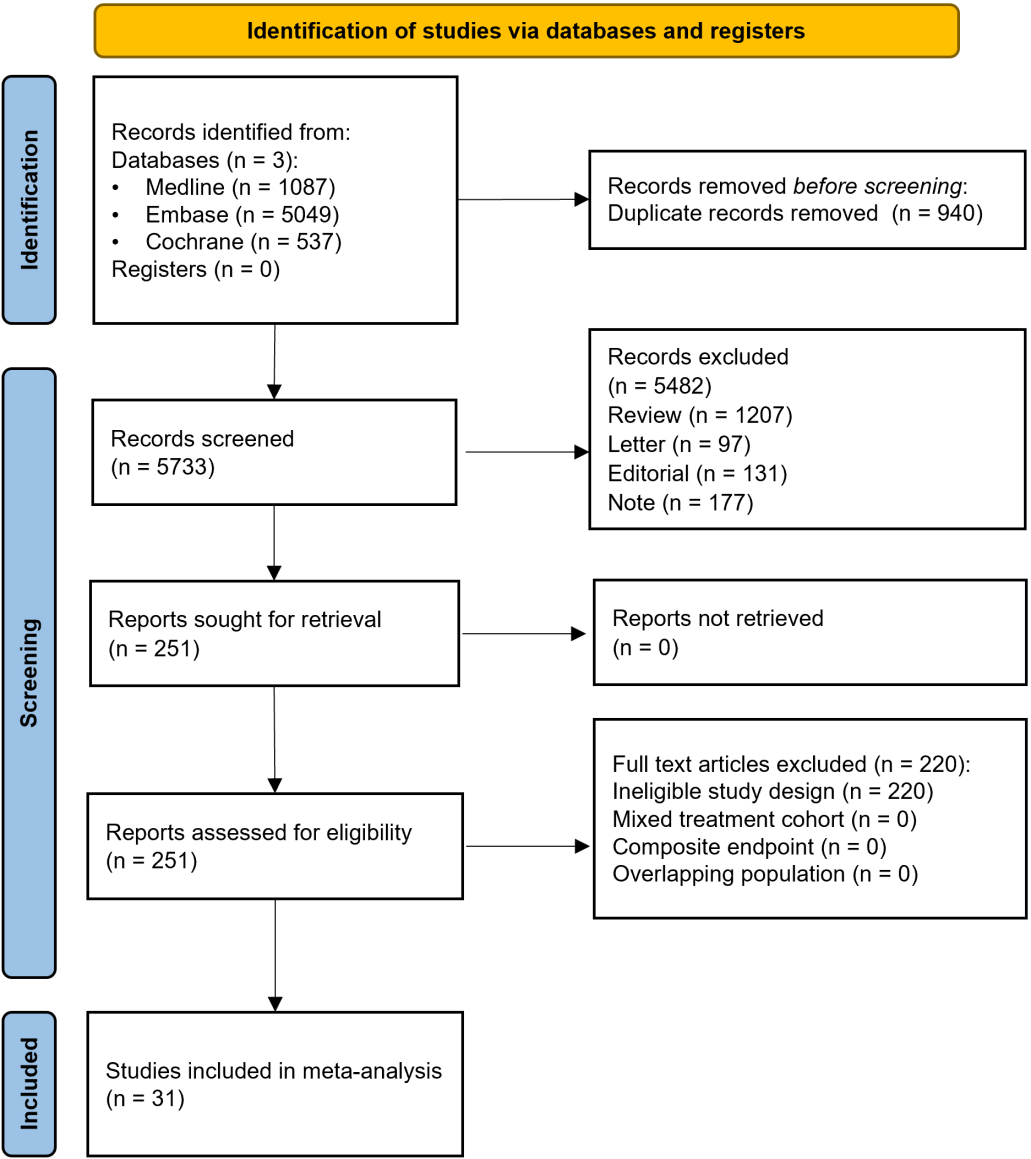
PRISMA 2020 flowchart illustrating the study selection process.

Using the specified search key, we obtained a total of 6,673 articles from the databases accessed (**Figure 1**). After the selection process, 31 articles met our eligibility criteria.

### Baseline characteristics of studies included

The baseline characteristics of the retrieved articles are shown in **Table 1**. All articles included ICI-treated mUC either in the lower or the upper urinary tract (UTUC). The median age of patients at baseline ranged from 65 to 74 years, with a female ratio from 13 to 38%. The median percentage of UTUC cases was 38.5%. Nineteen studies used pembrolizumab, and four applied atezolizumab. Eight articles used other or more than one ICI drugs. Four articles reported results from prospective studies, and twentyseven were retrospective. Seventeen studies collected data from multicenter databases, and fourteen articles were singlecenter. The median rate of patients with performance status ECOG >1 was 14%. Nineteen articles included patients who received second-line ICI therapy, whereas two articles used first-line ICI. The remaining ten articles either provided no information on the line of therapy or included both first-line and second-line ICI-treated patients.

**Table 1:** Basic characteristics of articles included.

Author (year)	Study site/center/type	Nr. of pts. (female %)	Type of treatment	Age (median, range)	UTUC %	Follow-up in months (median, range)	ECOG 2-4 (%)	Line of therapy	Biomarker - Outcome
Bamias (2023) (49)	Italy/Multi/P	936 (22)	Atezo	68 (61 - 74)	23	12.6	N/A	2L	NLR - OS
Brown (2021) (50)	USA/Single/R	53 (15)	Mixed	70 (32 - 86)	N/A	27.1	11	Mixed	CRP - OS
Kouchkovsky (2021)	USA/Single/R	119 (35)	Mixed	71 (65 - 77)	24	6.3	21	N/A	NLR - OS, PFS
Fornarini (2021) (51)	Italy/Multi/P	267 (17)	Atezo	69 (62 – 74)*	20	9.5	5	2L	NLR - OS, PFS
Fujiwara (2021) (52)	Japan/Single/R	74 (26)	Pembro	69 (61 - 73)*	51	8.5 (3.5 – 15.7)*	10	2L	NLR, CRP, LDH - OS
Fukushima (2020) (53)	Japan/Single/R	28 (32)	Pembro	74 (70 - 82)*	32	6 (3 – 18)*	11	2L	CRP - OS, PFS
Furubayashi (2021) (54)	Japan/Multi/R	105 (29)	Pembro	72 (67 - 77)*	39	8.4 (4.1 – 15.7)*	10	2L	LDH - OS
Isobe (2021) (55)	Japan/Single/R	94 (18)	Pembro	72 (47 - 85)	N/A	13.6	1	2L	NLR, CRP - OS
Ito (2021) (56)	Japan/Multi/R	755 (25)	Pembro	72 (63 - 77)	N/A	7.2	20	2L	NLR - OS
Khaki (2021) (57)	USA-EU/Multi/R	357 (27)	Mixed	71 (32 - 93)	13	22	29	1L	NLR - OS
Klümper (2021) (43)	Germany/Multi/R	154 (26)	Mixed	68 (43 - 88	19	N/A	14	Mixed	CRP - OS, PFS
Kobayashi (2021) (58)	Japan/Multi/R	463 (23)	Pembro	71 (31 - 88)	39	10.2	19	Mixed	NLR - OS
Kurushina (2022) (59)	Japan/Single/R	54 (32)	Pembro	70** (51 - 81)	N/A	N/A	N/A	2L	NLR - OS, PLR - OS, PFS
Miyama (2022) (60)	Japan/Single/R	50 (38)	Pembro	72 (70 -77)	46	N/A	N/A	2L	NLR - OS, PFS
Ogihara (2020) (61)	Japan/Single/R	78 (31)	Pembro	72** (46 -89)	45	7.42 (0.9 – 7.9)	N/A	2L	NLR - PFS
Park (2022) (62)	Korea/Multi/R	224 (28)	Mixed	68 (32 - 90)	42	10.5 (5.1 – 17.4)*	10	2L	NLR - OS
Pond (2021) (63)	Mixed/Multi/R	79 (N/A)	Mixed	74 (45 - 93)	N/A	N/A	28	1L	NLR - OS
Rijnders (2022) (64)	Netherlands/Single/P	71 (28)	Pembro	70 (29 - 85)	30	N/A	33	Mixed	NLR - OS, PFS
Shabto (2020) (65)	USA/Single/R	67 (21)	Mixed	69 (32 - 93)	N/A	N/A	12	Mixed	NLR, PLR - OS, PFS
Shimizu (2020) (66)	Japan/Single/R	27 (15)	Pembro	73 (52 - 82)	44	7 (1 – 20)	44	2L	NLR, CRP, PLR - OS, PFS
Sonpavde (2020) (67)	USA/Multi/P	405 (23)	Atezo	66 (32 - 89)	N/A	22.8 (19.2 – 30)	N/A	2L	NLR, LDH - OS
Taguchi (2021) (68)	Japan/Multi/R	150 (26)	Pembro	71 (66–76)	45	7.5 (4 – 14)*	12	Mixed	NLR, CRP - OS, PFS
Tamura (2019) (69)	Japan/Single/R	41 (30)	Pembro	70 (47 - 82)	54	6.2** (0.3 – 18)	15	2L	NLR, CRP - OS
Tomioka-Inagawa (2022)	Japan/Multi/R	160 (25)	Pembro	72 (69 - 78)*	31	10 (5 – 19)*	16	2L	NLR, CRP - OS
Tural (2021) (70)	Turkey/Multi/R	113 (13)	Atezo	65 (37 – 86)	13	23.5	N/A	Mixed	NLR - OS
Uchimoto (2021) (71)	Japan/Multi/R	212 (29)	Pembro	72 (66 - 78)*	39	11.7	N/A	2L	NLR - OS
Une (2022) (72)	Japan/Single/R	200 (31)	Pembro	71 (38 - 94)	45	13.3 (0 – 183)	11	Mixed	CRP, LDH - OS
Váradi (2022)	Hungary/Multi/R	210 (31)	Mixed	70 (29- 89)	12	10.2 (0 -68.7)	12	Mixed	NLR, CRP, LDH - OS, PFS
Yamamoto (2021) (73)	Japan/Multi/R	121 (28)	Pembro	74 (50 - 86)	46	7.9 (0.8 – 55.9)	N/A	2L	NLR, CRP - OS
Yasuoka (2019) (74)	Japan/Single/R	40 (20)	Pembro	69 (44 - 83)	48	5.3 (1.4 -12.3)	14	2L	NLR, CRP, LDH - OS
Yoshida (2022) (75)	Japan/Multi/R	755 (25)	Pembro	72 (66 - 77)	56	N/A	20	2L	NA

Pembro, pembrolizumab; Atezo, atezolizumab; Single, Singlecenter; Multi, Multicenter; R, retrospective; P, prospective; OS, overall survival; PFS, progression-free survival *IQR, **mean, UTUC, upper tract urothelial carcinoma; ECOG, Eastern Cooperative Oncology Group.

### High pre-treatment NLR is associated with inferior OS and PFS

**Figure 2:**
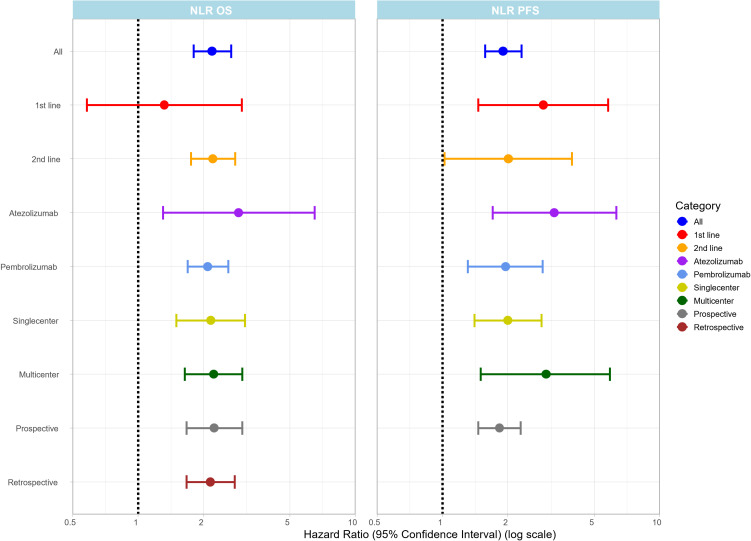
Summary plot showing pooled HR values (x-axis) with 95% CI for OS and PFS for NLR in different subgroups (y-axis). The forest plot for each subgroup is provided in the **Supplementary Material**.

Twenty articles provided information on NLR and OS. Pre-treatment high NLR was associated with worse OS both in univariate (HR: 2.19; 95%CI: 1.80-2.68) (**Figure 2**) and multivariate analyses (HR: 1.77; 95%CI: 1.61-1.94) (**Supplementary Figure 1**). High pre-treatment NLR was associated with poor PFS both in univariate (HR: 1.90; 95%CI: 1.57-2.31) (**Figure 2**) and multivariate analysis (HR: 1.77; 95%CI: 1.16-2.71) (**Supplementary Figure 2**).

Subgroup analysis of therapy lines revealed that high pre-treatment NLR was associated with worse OS rates in the second-line (12 articles) (HR:2.21 95%CI: 1.75-2.80) and the mixed-line (5 articles) (HR:3.03 95%CI: 1.67-5.52) ICI settings but no significant association was found in the first-line setting (3 articles) (HR:1.32 95%CI: 0.58-3.00);. Furthermore, subgroup analysis by ICI drug type revealed that NLR was associated with worse OS rates both in the pembrolizumab (12 articles) (HR: 2.09; 95%CI: 1.69-2.60) (**Figure 2**) and in the atezolizumab (4 articles) (HR: 2.90; 95%CI: 1.30-6.49) (**Figure 2**) treatment groups. In an additional subgroup analysis, OS rate remained consistently associated with NLR regardless of study design, with an HR of 2.24 (95%CI: 1.67-3.01) (**Figure 2**) for prospective studies (3 articles) and an HR of 2.15 (95%CI: 1.67-2.78) (**Figure 2**) for retrospective studies (17 articles). In addition, singlecenter studies had results similar to those of multicenter studies, with singlecenter studies (8 articles) giving an HR of 2.16 (95%CI: 1.50-3.10) (**Figure 2**) and multicenter studies (12 articles) an HR of 2.23 (95%CI: 1.64-3.02) (**Figure 2**). Three articles provided information on NLR and ORR, with a pooled ORR of 1.66 (95%CI: 0.47-5.89) (**Supplementary Figure 3**).

### High pre-treatment CRP levels are associated with inferior OS and PFS

**Figure 3:**
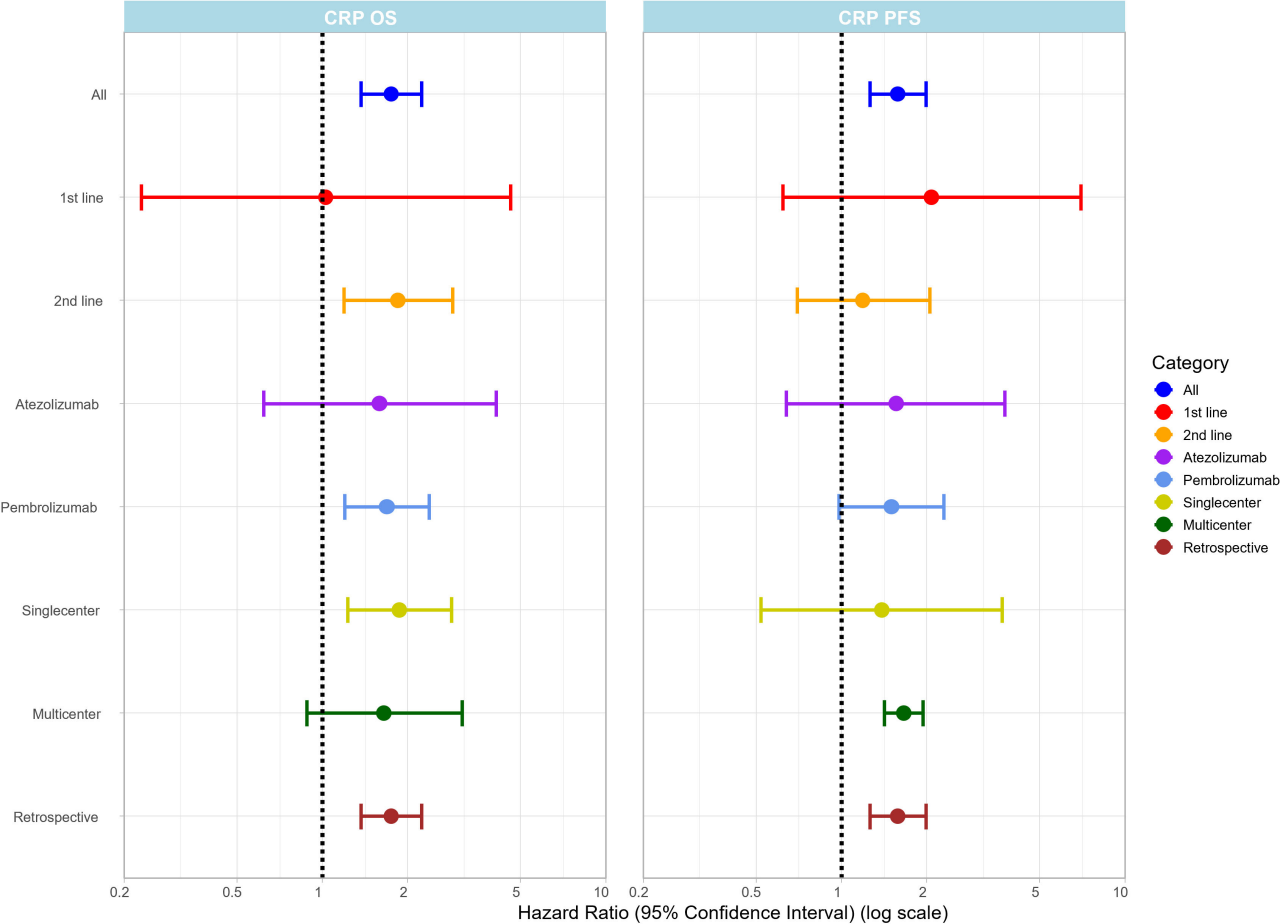
Summary plot of pooled HR values (x-axis) with 95% CI for OS and PFS for the CRP based on in different subgroups on the (y-axis). The forest plot for each subgroup is provided in the **Supplementary Material**.

Eleven articles provided information on pre-treatment serum CRP levels. High pre-treatment CRP levels were associated with lower OS rates in both the univariate (HR: 1.75; 95%CI: 1.37-2.24) (**Figure 3**) and multivariate (HR: 1.66; 95%CI: 1.18-2.33) (**Supplementary Figure 4**) analyses. Similarly, poor PFS was associated with elevated pre-treatment CRP levels (HR: 1.58; 95%CI: 1.26-1.99) (**Figure 3**).

Our subgroup analysis revealed that in the second-line ICI setting (7 articles), high pre-treatment CRP was associated with worse OS rates (HR: 1.85; 95% CI: 1.19-2.88) (**Figure 3**). Furthermore, CRP was also associated with worse OS rates in the pembrolizumab (9 articles) (HR: 1.69; 95%CI: 1.20-2.38) (**Figure 3**) treatment group, whereas for atezolizumab, the two available studies did not allow a statistical evaluation. In addition, CRP levels were associated with poor OS in singlecenter (7 articles) (HR: 1.87; 95%CI: 1.23-2.86) (**Figure 3**), but not in multicenter studies (4 articles).

### High pre-treatment PLR is associated with inferior OS and PFS

**Figure 4:**
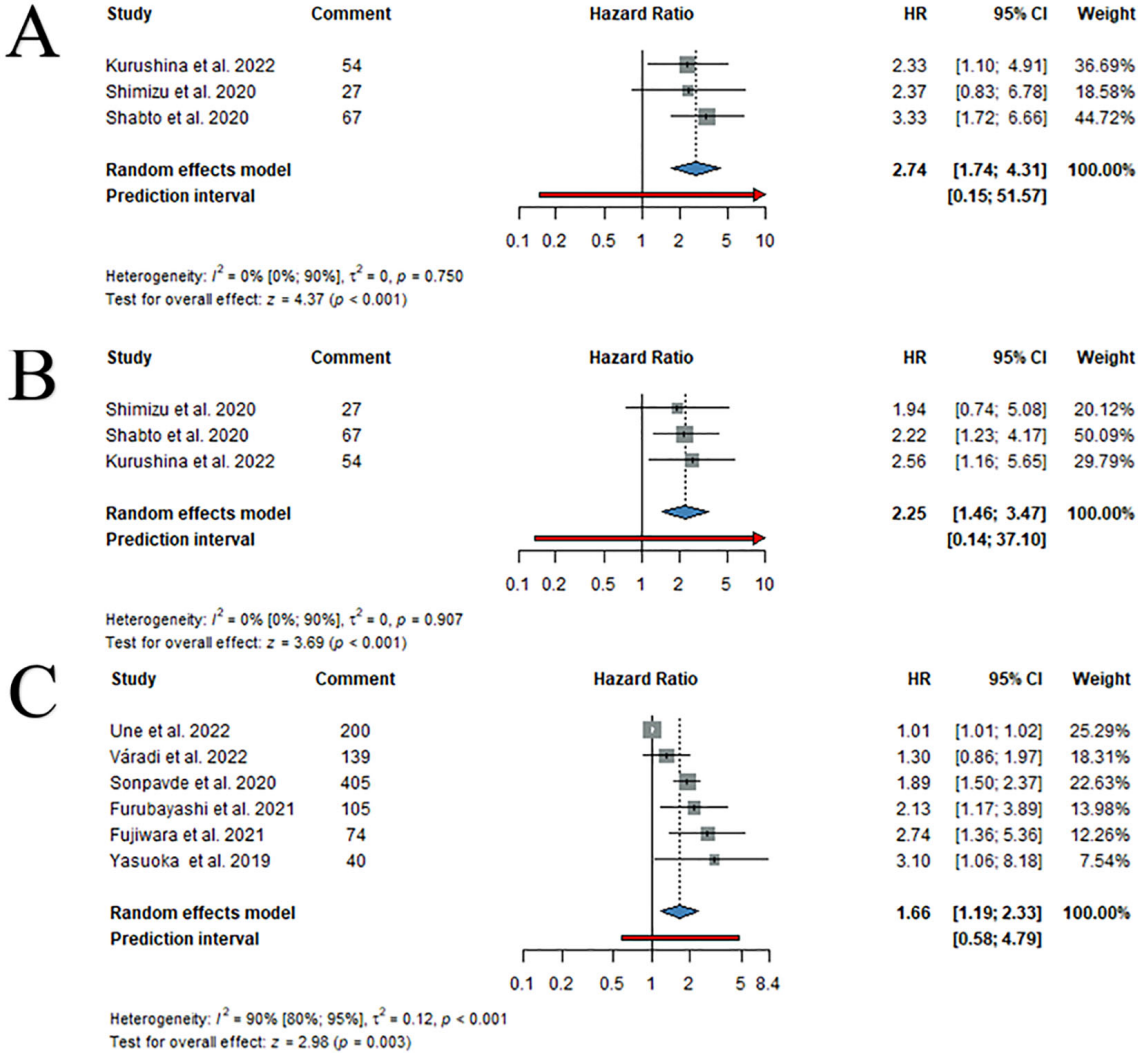
Forest plots of pooled univariate HR values with 95% CI for PLR OS **(A)**, PLR PFS **(B)** and LDH OS **(C)**.

Three articles provided data on PLR and survival endpoints (OS, PFS). In univariate analysis, high pre-treatment PLR was associated with shorter OS (HR: 2.74; 95%CI: 1.74-4.31) (**Figure 4**) and PFS (HR: 2.25; 95%CI: 1.46-3.47) (**Figure 4**). Subgroup analyses were not possible due to the low number of available articles.

### High pre-treatment LDH levels are associated with inferior OS and PFS

Six articles provided information on LDH and OS. In univariate analysis, high pre-treatment LDH was associated with lower OS (HR: 1.66; 95%CI: 1.19-2.33) (**Figure 4**). Furthermore, LDH was also associated with worse OS rates for second-line ICI therapy (HR: 1.90; 95%CI: 1.33-2.73) (**Supplementary Figure 5**) treatment. Further subgroup analyses were limited due to the low number of available articles.

### Risk of bias assessment and level of evidence

Traffic light plot with risk of bias assessment resulted in a low risk of bias in 23 of 31 articles, while 6 articles presented severe concerns and 2 articles had a high risk of bias. (**Supplementary Table 3**)

## Discussion

In this systematic review and meta-analysis, we evaluated the association between various blood-based biomarkers and the efficacy of ICI treatment in mUC patients, using ORR, OS, and PFS as endpoints. NLR, CRP, PLR, and LDH are widely used and easy-to-reach blood-based biomarkers that reflect the systemic inflammatory status.

Over the past few years, several articles have provided information on these biomarkers in different tumor entities and treatment options. In 2021, Yanagisawa et al. performed a meta-analysis focusing on the prognostic significance of pre-treatment parameters in mUC patients who underwent pembrolizumab therapy. They found that high levels of NLR and CRP were associated with inferior OS. However, their analysis was restricted only to pembrolizumab, and at the time of publication, only a limited number of articles were available for NLR (n=5) and CRP (n=2) (33). Since then, a large number of articles have been published, providing an opportunity to perform a more detailed analysis. In this meta-analysis, we summarized data of 31 articles focusing on the blood-based soluble biomarkers of inflammation in 6,412 mUC patients who underwent different types of ICI treatments.

A higher neutrophil count reduces CD8+ T-cell count, suggesting that a low NLR value is a favorable predictor of immunity in a healthy host (34). Thus, NLR can be considered a potential marker of ICI sensitivity. We collected NLR data from 20 articles with an overall number of 3,886 mUC patients and found that those with high NLR levels had a 119% higher risk of death and a 90% higher risk of progression. Furthermore, 1,504 patients treated with atezolizumab and 1,540 patients treated with pembrolizumab with high NLR levels had a 190% and 109% increased risk of death, respectively. We found that the study design (retrospective *vs.* prospective and singlecenter *vs.* multicenter) did not affect the findings on NLR. The question of whether NLR is prognostic or predictive of therapy remains unanswered. Rossi et al. found that mUC patients with elevated NLR had significantly worse PFS and OS on platinum-based chemotherapy (35). In a further meta-analysis in the chemotherapy subgroup with elevated NLR a 44% higher risk of death was detected (36). For mUC patients treated third-line enfortumab vedotin, two publications found no significant association between NLR and OS or radiographic progression (37, 38). In contrast, we found a much stronger association between NLR and OS as well as PFS, suggesting that high NLR values were more associated with patient outcomes in ICI-treated than in platinum or enfortumab vedotin-treated patients. For this reason, NLR can be used to select patients who are less responsive to immunotherapy. In the neoadjuvant setting, authors of the SWOG 8710 prospective trial concluded that NLR was not a reliable predictor of OS (39). On the basis of the findings, it appears that NLR is a good pre-treatment predictor in later-line settings regardless of treatment modality.

CRP is an acute-phase protein and an indicator of inflammatory status. Pre-treatment CRP has been widely used in different treatment settings. In UC, CRP has been investigated in almost all stages and treatment modalities with promising results. Tekemura et al. found that patients with advanced BC treated with cisplatin who had high pre-chemo CRP had a 73% higher risk of worse OS (40). We observed similar results with patients with high CRP levels having a 75% increased risk of worse OS. Moving on to therapeutic prediction, we collected PFS data and observed a 58% higher risk of progression in patients treated with ICI. Eggers et al. found that BC patients treated with gemcitabine plus platinum had significantly lower median PFS when their pre-treatment CRP levels were elevated (41). Moreover, dynamic changes in CRP levels during ICI treatment have been investigated as a potential indicator of treatment response in mUC (42–45). A decrease in CRP levels after initiation of ICI therapy has been correlated with improved outcomes, indicating a favorable treatment response. Conversely, persistently elevated or increasing CRP levels during treatment may indicate resistance to therapy and poorer prognosis. In addition, Tomisaki et al. and Klümper et al. categorized patients into 1) ‘CRP-responders’, whose CRP levels decreased to normal levels after therapy, 2) ‘non-responders’, whose CRP levels remained high after therapy, and 3) ‘flare responders’, whose CRP levels had doubled compared to baseline within one month of starting ICI therapy (CRP flare), followed by a subsequent decrease to below the baseline within three months (43, 45). They found 12-16% of patients in the flare response group with a favorable ORR of 69-75% (42, 43, 45),. These findings implicates that CRP holds promise as a dynamic on-treatment monitoring biomarker that could allow an early therapy switch in non-responder patents thereby improving patient management during ICI treatment. Therefore, pre-treatment CRP levels appear to be prognostic in both platinum and ICI treatment, and also its kinetics during therapy CRP holds promise as an on-treatment monitoring biomarker for ICI treatment.

LDH is a rather nonspecific serum marker mainly reflecting tumor aggressiveness, hypoxia, metabolic deteriorations, and tumor lysis. While its elevated levels were associated with poor patients’ prognosis, its clinical utility may be challenging due to the fact that various confounding factors such as liver dysfunction or tumor burden are known to influence its serum levels (46). Therefore, its prognostic and predictive value should be tested in independent studies applying multivariate analyses in order to exclude the influence of possible confounding factors.

Recently, PLR has also been linked to the prediction of various human malignancies. It is well-studied that platelets play an inevitable role in tumor cell survival and metastasis formation (47). Wang et al. conducted a meta-analysis of surgically-treated BC patients and found a significant 26% higher risk of death in patients with high pre-treatment PLR values (48). However, in the context of subsequent therapeutic modalities and especially ICI, only three articles have recently been published that concordantly conclude that patients with high pre-treatment PLR levels have a strongly increased risk of death (274%) and progression (225%) during ICI therapy. Therefore, PLR may serve as a potential predictor of patient prognosis during ICI therapy. Due to the limited number of publications and available PLR data, our findings should be considered as an encouraging signal and serve as a basis for future validation in larger studies.

### Strengths and limitations

The strength of this study is the high number of articles included, which allowed us to perform relevant subgroup analyses. We used no restrictions to ICI drugs as in former studies and included all relevant outcomes. Furthermore, this is the first meta-analysis on PLR in ICI-treated UC. Nonetheless, we also faced some limitations. Most studies were retrospective and potentially introducing bias, and only a few were prospective. The limited number of studies on certain biomarkers, such as PLR and LDH, restricted subgroup analyses and weakened the strength of conclusions on these markers. While most studies had a low risk of bias, two out of thirty one had severe concerns or high risk. Heterogeneity varies across different outcomes; however, subgroup analysis and the high number of included studies in our meta-analysis contribute to its reduction. A further limitation arises from the lack of patient level data, which prevented the performance of multivariate analyses. While we collected results from multivariate analyses, the models across different studies included various parameters, limiting the comparability between them.

### Implications for practice, research and policymakers

If validated, these biomarkers can assist in selecting the appropriate therapy, particularly in the light of the increasing complexity of treatment regimens. In addition, they can also help patient stratification, treatment monitoring thus preventing the use of ineffective treatments, thereby minimizing unnecessary side effects. Therefore, incorporating these biomarkers into future risk stratification models (e.g. nomograms) could assist clinicians in developing more personalized treatment strategies. Furthermore, the assessed markers are widely available form a routine laboratory blood test and are thus easy-to-implement at low costs in everyday clinical practice.

## Conclusions

We conclude that high pre-treatment inflammatory biomarkers such as NLR, CRP, LDH, and PLR hold promise as reliable prognostic biomarkers in ICI therapy. Therefore, these biomarkers are good candidates for inclusion in future risk stratification models for mUC therapy. However, a prospective biomarker-based studies are needed to strengthen the evidence of our findings and extend the analysis of other treatment options in this rapidly evolving field.

## Data Availability

The original contributions presented in the study are included in the article/**Supplementary Material**. Further inquiries can be directed to the corresponding author.
