# Supplementary material for: Prognostic and predictive value of pre-treatment blood-based inflammatory biomarkers in patients with urothelial carcinoma treated with immune checkpoint inhibitors: a systematic review and meta-analysis
Source: Front Immunol. 2025 Mar 17;16:1554048. doi: 10.3389/fimmu.2025.1554048 (PMC11955586; doi:10.3389/fimmu.2025.1554048)
Supplement: Supplementary file 1 [file DataSheet1.docx]

**Supplementary material**

Prognostic and predictive value of pre-treatment blood-based inflammatory biomarkers in patients with urothelial carcinoma treated with immune checkpoint inhibitors: a systematic review and meta-analysis

**Authors**

Ádám Széles^1,2^, András Kubik ^1,2^, Szilárd Váncsa^2,3,4^, Viktor Grünwald^5^, Boris Hadaschik^5^, Nándor Ács^2,6^, Péter Hegyi^2,3,4^, Péter Nyirády^1,2*^, Tibor Szarvas^1,2,5*^

*authors contributed equally

**Affiliations:**

1. Department of Urology, Semmelweis University, Budapest, Hungary
2. Center for Translational Medicine, Semmelweis University, Budapest, Hungary
3. Institute for Translational Medicine, Medical School, University of Pécs, Pécs, Hungary
4. Institute of Pancreatic Diseases, Semmelweis University, Budapest, Hungary
5. Department of Urology, University of Duisburg-Essen and German Cancer Consortium (DKTK)-University Hospital Essen, Essen, Germany
6. Department of Obstetrics and Gynecology, Semmelweis University, Budapest, Hungary

**Corresponding author**

Tibor Szarvas, PhD, DSc

Department of Urology, Semmelweis University, Üllői út 78/B. 1082 Budapest, Hungary

Department of Urology, University Duisburg-Essen, Hufelandstr 55, 45147, Essen, Germany

Tel.: +36-1-210-0280

Fax.: +36-1-210-0305

E-mail: [szarvas.tibor@semmelweis.hu](mailto:szarvas.tibor@semmelweis.hu); [tibor.szarvas@uk-essen.de](mailto:tibor.szarvas@uk-essen.de)

**TABLE OF CONTENT**

**Supplementary Table 1.** PRISMA checklist

**Supplementary Table 2.** Inclusion and exclusion criteria for the included articles

**Supplementary Table 3.** Traffic light plot representing risk of bias assessment

**Supplementary Table 4.** Grading of evidence based on GRADEpro^TM^

**Supplementary Figure 1.** Forrest plot representing pooled multivariate analysis of overall survival, NLR biomarker

**Supplementary Figure 2.** Forrest plot representing pooled multivariate analysis of progression free survival, NLR biomarker

**Supplementary Figure 3.** Forrest plot representing objective response rates of NLR biomarker

**Supplementary Figure 4.** Forrest plot representing pooled multivariate analysis of overall survival, CRP biomarker

**Supplementary Figure 5.** Forrest plot representing subgroup analysis based on line of therapy pooled univariate analysis of overall survival, LDH biomarker

**Supplementary Figure 6.** Forrest plot representing pooled univariate analysis of overall survival, NLR biomarker

**Supplementary Figure 7.** Forrest plot representing pooled univariate analysis of progression free survival, NLR biomarker

**Supplementary Figure 8.** Forrest plot representing subgroup analysis based on line of therapy pooled univariate analysis of overall survival, NLR biomarker

**Supplementary Figure 9.** Forrest plot representing subgroup analysis based on type of therapy pooled univariate analysis of overall survival, NLR biomarker

**Supplementary Figure 10.** Forrest plot representing subgroup analysis based on type of center pooled univariate analysis of overall survival, NLR biomarker

**Supplementary Figure 11.** Forrest plot representing subgroup analysis based on study design pooled univariate analysis of overall survival, NLR biomarker

**Supplementary Figure 12.** Forrest plot representing subgroup analysis based on line of therapy pooled univariate analysis of progression free survival, NLR biomarker

**Supplementary Figure 13.** Forrest plot representing subgroup analysis based on type of therapy pooled univariate analysis of progression free survival, NLR biomarker

**Supplementary Figure 14.** Forrest plot representing subgroup analysis based on type of center pooled univariate analysis of progression free survival, NLR biomarker

**Supplementary Figure 15.** Forrest plot representing subgroup analysis based on study design pooled univariate analysis of progression free survival, NLR biomarker

**Supplementary Figure 16.** Forrest plot representing pooled univariate analysis of overall survival, CRP biomarker

**Supplementary Figure 17.** Forrest plot representing pooled univariate analysis of progression free survival, CRP biomarker

**Supplementary Figure 18.** Forrest plot representing subgroup analysis based on line of therapy pooled univariate analysis of overall survival, CRP biomarker

**Supplementary Figure 19.** Forrest plot representing subgroup analysis based on type of therapy pooled univariate analysis of overall survival, CRP biomarker

**Supplementary Figure 20.** Forrest plot representing subgroup analysis based on type of center pooled univariate analysis of overall survival, CRP biomarker

**Supplementary Figure 21.** Forrest plot representing subgroup analysis based on study design pooled univariate analysis of overall survival, CRP biomarker

**Supplementary Figure 22.** Forrest plot representing subgroup analysis based on line of therapy pooled univariate analysis of progression free survival, CRP biomarker

**Supplementary Figure 23.** Forrest plot representing subgroup analysis based on type of therapy pooled univariate analysis of progression free survival, CRP biomarker

**Supplementary Figure 24.** Forrest plot representing subgroup analysis based on type of center pooled univariate analysis of progression free survival, CRP biomarker

**Supplementary Figure 25.** Forrest plot representing subgroup analysis based on study design pooled univariate analysis of progression free survival, CRP biomarker

**Supplementary text 1.** Search key

**Supplementary Table 1.** PRISMA checklist [1]

| **Section and topic** | **Item #** | **Checklist item** | **Location where item is reported** |  |
| --- | --- | --- | --- | --- |
| **Title** | | | | |
| Title | 1 | Identify the report as a systematic review. | 1 |  |
| **Abstract** | | | | |
| Abstract | 2 | See the PRISMA 2020 for Abstracts checklist (table 2). | 3 |  |
| **Introduction** | | | | |
| Rationale | 3 | Describe the rationale for the review in the context of existing knowledge. | 4 |  |
| Objectives | 4 | Provide an explicit statement of the objective(s) or question(s) the review addresses. | 5 |  |
| **Methods** | | | | |
| Eligibility criteria | 5 | Specify the inclusion and exclusion criteria for the review and how studies were grouped for the syntheses. | 5-6 |  |
| Information sources | 6 | Specify all databases, registers, websites, organisations, reference lists and other sources searched or consulted to identify studies. Specify the date when each source was last searched or consulted. | 6 |  |
| Search strategy | 7 | Present the full search strategies for all databases, registers and websites, including any filters and limits used. | 6 |  |
| Selection process | 8 | Specify the methods used to decide whether a study met the inclusion criteria of the review, including how many reviewers screened each record and each report retrieved, whether they worked independently, and if applicable, details of automation tools used in the process. | 6 |  |
| Data collection process | 9 | Specify the methods used to collect data from reports, including how many reviewers collected data from each report, whether they worked independently, any processes for obtaining or confirming data from study investigators, and if applicable, details of automation tools used in the process. | 6 |  |
| Data items | 10a | List and define all outcomes for which data were sought. Specify whether all results that were compatible with each outcome domain in each study were sought (e.g. for all measures, time points, analyses), and if not, the methods used to decide which results to collect. | 6 |  |
|  | 10b | List and define all other variables for which data were sought (e.g. participant and intervention characteristics, funding sources). Describe any assumptions made about any missing or unclear information. | 6 |  |
| Study risk of bias assessment | 11 | Specify the methods used to assess risk of bias in the included studies, including details of the tool(s) used, how many reviewers assessed each study and whether they worked independently, and if applicable, details of automation tools used in the process. | 6 |  |
| Effect measures | 12 | Specify for each outcome the effect measure(s) (e.g. risk ratio, mean difference) used in the synthesis or presentation of results. | 6 |  |
| Synthesis methods | 13a | Describe the processes used to decide which studies were eligible for each synthesis (e.g. tabulating the study intervention characteristics and comparing against the planned groups for each synthesis (item #5)). | 6-7 |  |
|  | 13b | Describe any methods required to prepare the data for presentation or synthesis, such as handling of missing summary statistics, or data conversions. | 6-7 |  |
|  | 13c | Describe any methods used to tabulate or visually display results of individual studies and syntheses. | 6-7 |  |
|  | 13d | Describe any methods used to synthesise results and provide a rationale for the choice(s). If meta-analysis was performed, describe the model(s), method(s) to identify the presence and extent of statistical heterogeneity, and software package(s) used. | 6-7 |  |
|  | 13e | Describe any methods used to explore possible causes of heterogeneity among study results (e.g. subgroup analysis, meta-regression). | 6-7 |  |
|  | 13f | Describe any sensitivity analyses conducted to assess robustness of the synthesised results. | 6-7 |  |
| Reporting bias assessment | 14 | Describe any methods used to assess risk of bias due to missing results in a synthesis (arising from reporting biases). | 6-7 |  |
| Certainty assessment | 15 | Describe any methods used to assess certainty (or confidence) in the body of evidence for an outcome. | 6-7 |  |
| **Results** | | | | |
| Study selection | 16a | Describe the results of the search and selection process, from the number of records identified in the search to the number of studies included in the review, ideally using a flow diagram (see fig 1). | 8 |  |
|  | 16b | Cite studies that might appear to meet the inclusion criteria, but which were excluded, and explain why they were excluded. | 8 |  |
| Study characteristics | 17 | Cite each included study and present its characteristics. | 8 |  |
| Risk of bias in studies | 18 | Present assessments of risk of bias for each included study. | Suppl. |  |
| Results of individual studies | 19 | For all outcomes, present, for each study: (a) summary statistics for each group (where appropriate) and (b) an effect estimate and its precision (e.g. confidence/credible interval), ideally using structured tables or plots. | Figures |  |
| Results of syntheses | 20a | For each synthesis, briefly summarise the characteristics and risk of bias among contributing studies. | Suppl. |  |
|  | 20b | Present results of all statistical syntheses conducted. If meta-analysis was done, present for each the summary estimate and its precision (e.g. confidence/credible interval) and measures of statistical heterogeneity. If comparing groups, describe the direction of the effect. | 8-10 |  |
|  | 20c | Present results of all investigations of possible causes of heterogeneity among study results. | 8-10 |  |
|  | 20d | Present results of all sensitivity analyses conducted to assess the robustness of the synthesised results. | 8-10 |  |
| Reporting biases | 21 | Present assessments of risk of bias due to missing results (arising from reporting biases) for each synthesis assessed. | 8-10 |  |
| Certainty of evidence | 22 | Present assessments of certainty (or confidence) in the body of evidence for each outcome assessed. | 8-10 |  |
| **Discussion** | | | | |
| Discussion | 23a | Provide a general interpretation of the results in the context of other evidence. | 11 |  |
|  | 23b | Discuss any limitations of the evidence included in the review. | 11-12 |  |
|  | 23c | Discuss any limitations of the review processes used. | 11-12 |  |
|  | 23d | Discuss implications of the results for practice, policy, and future research. | 11-12 |  |
| **Other information** | | | | |
| Registration and protocol | 24a | Provide registration information for the review, including register name and registration number, or state that the review was not registered. | 5 |  |
|  | 24b | Indicate where the review protocol can be accessed, or state that a protocol was not prepared. | 5 |  |
|  | 24c | Describe and explain any amendments to information provided at registration or in the protocol. | 5 |  |
| Support | 25 | Describe sources of financial or non-financial support for the review, and the role of the funders or sponsors in the review. | 2 |  |
| Competing interests | 26 | Declare any competing interests of review authors. | 2 |  |
| Availability of data, code, and other materials | 27 | Report which of the following are publicly available and where they can be found: template data collection forms; data extracted from included studies; data used for all analyses; analytic code; any other materials used in the review. |  |  |

**Supplementary Table 2.** Inclusion and exclusion criteria for the included articles

| **Article** | **Inclusion criteria** | **Exclusion criteria** |
| --- | --- | --- |
| Bamias (2023) [42] | “a real- world patient population with locally advanced or metastatic UC or non- UC of the urinary tract. Patients were required to have ECOG PS≤2 and disease progression during or following 1–3 prior treatments. Patients with treated central nervous system (CNS) metastases, autoimmune disease, concomitant corticosteroids or renal impairment were eligible.” | “Patients were excluded from the current substudy if they had pure non- urothelial histology and/or had not received prior cisplatin or carboplatin- based chemotherapy.” |
| Brown (2021) [43] | “Inclusion criteria were biopsy-proven UC with spread to at least one metastatic site (including pelvic or retroperitoneal lymph nodes), prior treatment with a checkpoint inhibitor (either an anti–programmed cell death protein 1 [PD-1] or anti–PD-L1 monoclonal antibody) in any line of treatment at the Winship Cancer Institute of Emory University between the years of 2015 and 2018 and baseline CRP value available for analysis.” | No information |
| Kouchkovsky (2021) | “Patient eligibility criteria included: histologically confirmed UC, presence of locally advanced or metastatic disease, at least one dose of an ICI administered (including atezolizumab, pembrolizumab, nivolumab, durvalumab or avelumab), and available clinical, pathologic and imaging data prior to initiation of treatment. To be considered eligible for response assessment, a patient needed to have at least one scan following initiation of an ICI or clear evidence of clinical progression as assessed by the treating physician.” | “Patients who received an ICI for an indication other than aUC were excluded from this analysis.” |
| Fornarini (2021) [44] | “locally advanced (T4b Nany or TAny N2e3) or metastatic (M1) measurable and/or nonmeasurable urothelial or nonurothelial carcinoma of the urinary tract (bladder, ureter, urethra, or renal pelvis).20 Patients with renal impairment, treated central nervous system metastases, or stable controlled autoimmune disease were eligible for enrollment. All participants must have had ECOG PS 2 and disease progression during or following one (subsequently amended to up to three) prior platinum- or non-platinum-based treatments (or intolerance if they had received two or more cycles) for inoperable, locally advanced, or metastatic disease.” | No information |
| Fujiwara (2021) [45] | “The medical records of patients with previous platinum-based chemotherapy for the treatment of metastatic UC who received pembrolizumab as second- or later-line therapy at our institution between January 2018 and March 2020 were retrospectively reviewed.” | No information |
| Fukushima (2020) [46] | “All patients were histologically  diagnosed with urothelial carcinoma and progressed  after cisplatin-based or carboplatin-based chemotherapy.  All patients had at least one measurable lesion based  on the Response Evaluation Criteria in Solid Tumors  (RECIST), version 1.1” | No information |
| Furubayashi (2021) [47] | “advanced (metastatic or locally advanced) UC who received pembrolizumab after the failure of platinum-based chemotherapy at 6 institutions between January 2018 and June 2021. All patients were histopathologically diagnosed with UC and showed radiologically confirmed disease progression after platinum-based chemotherapy” | “20 patients were excluded from this study due to a lack of clinical data.” |
| Isobe (2021) [48] | “Criteria used for the  inclusion of patients were as follows: (1) had undergone surgery  or a biopsy of primary lesions, and enhanced computed  tomography (CT) was used for staging; (2) had a mUC diagnosis  with at least one cycle of first-line chemotherapy with  gemcitabine and cisplatin (1000 mg/m2 gemcitabine administered  on days 1, 8, and 15, and 70 mg/m2 cisplatin on days 1 or 2) or gemcitabine and carboplatin (1000 mg/m2 gemcitabine  administered on days 1 and 8, and carboplatin with an  area under the curve of 4–5 mg/mL/min on day 1) completed  after 4 weeks or more.” | - No information |
| Ito (2021) [49] | “UC who received pembrolizumab from August 2015 to December 2019 in a Japanese nationwide cohort” | No information |
| Khaki (2021) [50] | “Patients were included if they had aUC (locally advanced, unresectable, or metastatic) and were treated with ICIs for this indication” | “Patients were excluded from the current study if an ICI was given for alternate diagnosis or treatment setting (eg, [neo]adjuvant), they were treated with combinations or were on clinical trials, they received multiple lines of ICIs, or ICI start date or key covariates for modeling were missing.” |
| Klümper (2021) [37] | “Inclusion in the study cohort required measurement of CRP at least once within the first 30 days of treatment and at the time of first staging or clinical progression.” | No information |
| Kobayashi (2021) [51] | “patients with surgically unresectable, chemoresistant UC who received pembrolizumab were collected” | “5 patients were excluded due to missing data” |
| Kurashina (2022) [52] | “patients with previously treated UC who received pembrolizumab  treatment (200 mg pembrolizumab intravenously every 3 weeks) at  Saitama Cancer Center from January 2018 to December 2020.” | No information |
| Miyama (2022) [53] | “patients with platinum- resistant locally advanced or metastatic UC who received pembrolizumab in our institution” | No information |
| Ogihara (2020) [54] | “patients were treated  with pembrolizumab. All patients had histologically or  cytologically confirmed UC of the renal pelvis, ureter, or  bladder, had progression after cisplatin-based chemotherapy  for advanced diseases or recurrence within 1 year of  cisplatin-based adjuvant or neoadjuvant chemotherapy for  localized muscle invasive disease previously, had at least  one measurable lesion according to the Response Evaluation  Criteria in Solid Tumors (RECIST), version 1.1 [15],  and had an Eastern Cooperative Oncology Group performance  status (PS) score of 0, 1, or 2.” | “We excluded 1 patient  who did not visit a hospital after the initial treatment.” |
| Park (2022) [55] | “The inclusion criteria were as follows: diagnosis of histologically documented mUC of the renal pelvis, ureter, bladder, or urethra; advanced stage disease (recurrent, locoregionally advanced unresectable, or metastatic disease); treatment with ICIs after failure of platinum-based chemotherapy; and ade- quate clinical information and survival follow-up.” | “Patients were excluded from the current study if the tumor was pure non-urothelial type histologically, patients did not receive platinum-based chemotherapy, an ICI was administered in combination with cytotoxic chemotherapy, patients had serious medical conditions that could lead to deterioration of general condition or death independently of cancer, or essential covariates for prognostic modeling were missing.” |
| Pond (2021) [56] | “All patients enrolled had histopathologi- cal and radiologic confirmation of locally advanced unresectable or metastatic urothelial carcinoma.” | No information |
| Rijnders (2022)[57] | “Patients with locally advanced or mUC of the bladder or upper urinary tract with an indication for pembrolizumab were included in a phase II prospective biomarker discovery study (NCT03263039), and treated as described previously (pembrolizumab, 200 mg intravenously, 3-weekly” | No information |
| Shabto (2020) [58] | “UC patients treated with PD-1 or PD-L1 inhibitors at Winship Cancer Institute of Emory University between 2015 and 2018” | No information |
| Shimizu (2020) [59] | “patients who received pembrolizumab for  chemotherapy-resistant advanced mUC in Nara medical university hospital were initially enrolled in this study.” | “Two patients were excluded because of insufficient radiographic and laboratory examination,” |
| Sonpavde (2020) [60] | No information | No information |
| Taguchi (2021) [61] | “patients with advanced (locally-advanced or metastatic) UC treated with pembrolizumab at our seven affiliate institutions (five university hospitals and two tertiary referral hospitals) between January 2018 and July 2020” | “Excluded due to missing data (n=26)  Lack of AGR (n=7)  Lack of NLR (n=14)  Lack of of both (n=5)” |
| Tamura (2019) [62] | “patients with  advanced UC who were treated with pembrolizumab after  failure of platinum-based chemotherapy at our institution  from January 2018 to April 2019.” | No information |
| Tomioka-Inagawa (2022) | “All participants had previously received platinum-based chemotherapy and then subsequently their UC progressed” | No information |
| Tural (2021) [63] | “The decision to include the patients in the Expanded-  Access Program was made by the attending physician on  an individual basis.” | No information |
| Uchimoto (2021) [64] | “Inclusion criteria to the present study were as follows: patients who had one more measurable metastatic sites according to the response evaluation criteria in solid tumors (RECIST) version 1.1 (defined measurable lesion of ≥10 mm using spiral CT scan) [4] and had at least one radiographic examination during their follow-up; patients who had a blood examination at the initiation of pembrolizumab treatment; and patients who had no clinical record of comorbidities of immune disease, anticancer medications, and steroids at the initiation of pembrolizumab treatment.” | No information |
| Une (2022) [65] | “patients with inoperable (cT4 or lymph node metastasis) and/or metastatic UC of the bladder or UT were treated at a single, designated cancer center between December 2002 and December 2021” | “Of the 247 patients, 47 were excluded owing to missing data, which was required to calculate the CONUT score (n = 30) or missing CT imaging studies (n = 17).” |
| Váradi (2022) | “Eligible patients for inclusion were adults (≥ 18 years) with a confirmed diagnosis of advanced or metastatic urothelial tract malignancy, who received at least one cycle of ICI therapy (pembrolizumab or atezolizumab) as first- or second-line treatment between 01/2017 and 12/2021.” | “Patients with non-urothelial histology and those who were treated within clinical trials were excluded” |
| Yamamoto (2021) [66] | “patients with advanced UC that recurred or progressed after  platinum-based chemotherapy, and who received pembrolizumab  at 13 institutions of the Yamaguchi Uro-Oncology  Group and Kumamoto University between 2015 and 2019.” | No information |
| Yasuoka (2019) [67] | “patients with previous platinum-based chemotherapy for the treatment of metastatic UC who received pembrolizumab as second or later line therapy at our institution between January and October 2018 were retrospectively reviewed.” | No information |
| Yoshida (2022) [68] | “755 cases with surgically  unresectable, chemoresistant UC receiving pembrolizumab  were used for this study” | “we excluded  cases with visceral metastasis (defined as liver, lung, bone,  any non-lymph node, or soft tissue metastasis [1]) where  the specific-metastatic site of each case was unknown.” |

**Supplementary Table 3.** Traffic light plot representing risk of bias assessment


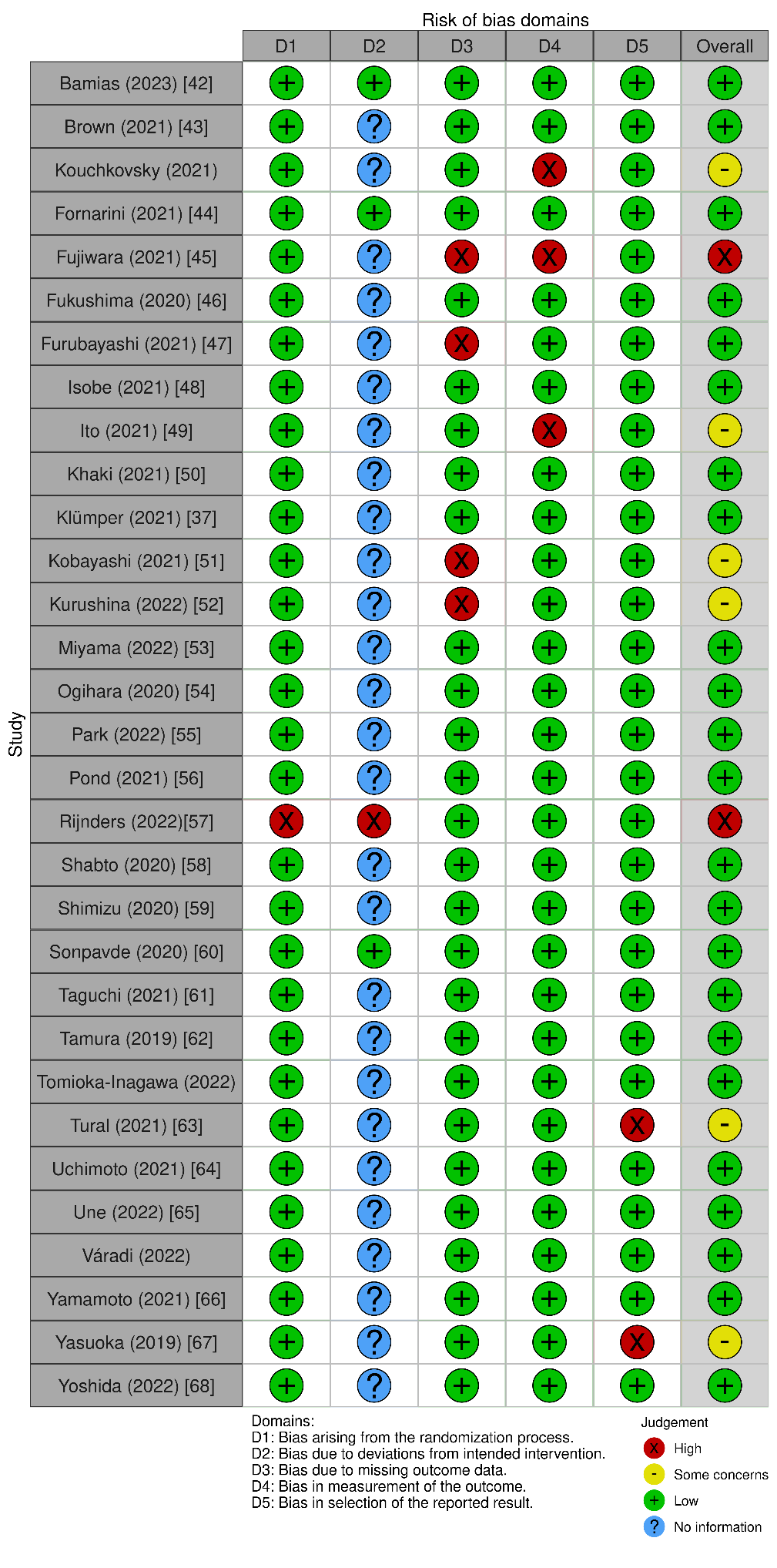


**Supplementary Table 4.** Grading of evidence based on GRADEpro^TM^

| **Certainty assessment** | | | | | | | **№ of patients** | | **Effect** | | **Certainty** | **Importance** |
| --- | --- | --- | --- | --- | --- | --- | --- | --- | --- | --- | --- | --- |
| **№ of studies** | **Study design** | **Risk of bias** | **Inconsistency** | **Indirectness** | **Imprecision** | **Other considerations** | **high NLR levels** | **low NLR levels** | **Relative (95% CI)** | **Absolute (95% CI)** |  |  |
| **NLR biomarker OS (assessed with: HR)** | | | | | | | | | | | | |
| 20 | Observational and prospective studies | not serious | not serious | not serious | not serious | none |  |  | **HR 2.19** (1.80 to 2.68) [New outcome] | **-- per 1 000** (from -- to --) | ⨁⨁⨁◯ Moderate | IMPORTANT |
|  |  |  |  |  |  |  | - | 0.0% |  | **-- per 1 000** (from -- to --) |  |  |
| **NLR biomarker PFS (assessed with: HR)** | | | | | | | | | | | | |
| 7 | Observational and prospective studies | not serious | not serious | not serious | not serious | none |  |  | **HR 1.90** (1.57 to 2.31) [New outcome] | **-- per 1 000** (from -- to --) | ⨁⨁⨁◯ Moderate | IMPORTANT |
|  |  |  |  |  |  |  | - | 0.0% |  | **-- per 1 000** (from -- to --) |  |  |

| **Certainty assessment** | | | | | | | **№ of patients** | | **Effect** | | **Certainty** | **Importance** |
| --- | --- | --- | --- | --- | --- | --- | --- | --- | --- | --- | --- | --- |
| **№ of studies** | **Study design** | **Risk of bias** | **Inconsistency** | **Indirectness** | **Imprecision** | **Other considerations** | **high CRP levels** | **low CRP levels** | **Relative (95% CI)** | **Absolute (95% CI)** |  |  |
| **CRP biomarker OS (assessed with: HR)** | | | | | | | | | | | | |
| 11 | observational studies | not serious | not serious | not serious | not serious | none |  |  | **HR 1.75** (1.37 to 2.24) [New outcome] | **-- per 1 000** (from -- to --) | ⨁⨁⨁◯ Moderate | IMPORTANT |
|  |  |  |  |  |  |  | - | 0.0% |  | **-- per 1 000** (from -- to --) |  |  |
| **CRP biomarker PFS (assessed with: HR)** | | | | | | | | | | | | |
| 6 | observational studies | not serious | not serious | not serious | not serious | none |  |  | **HR 1.58** (1.26 to 1.99) [New outcome] | **-- per 1 000** (from -- to --) | ⨁⨁⨁◯ Moderate | IMPORTANT |
|  |  |  |  |  |  |  | - | 0.0% |  | **-- per 1 000** (from -- to --) |  |  |

| **Certainty assessment** | | | | | | | **№ of patients** | | **Effect** | | **Certainty** | **Importance** |
| --- | --- | --- | --- | --- | --- | --- | --- | --- | --- | --- | --- | --- |
| **№ of studies** | **Study design** | **Risk of bias** | **Inconsistency** | **Indirectness** | **Imprecision** | **Other considerations** | **high LDH** | **low LDH** | **Relative (95% CI)** | **Absolute (95% CI)** |  |  |
| **LDH biomarker OS (assessed with: HR)** | | | | | | | | | | | | |
| 6 | observational studies | not serious | not serious | not serious | not serious | none | -  - | 0.0%  0.0% | **HR 1.66** (1.19 to 2.33) [New outcome] | **-- per 1 000** (from -- to --) | ⨁⨁⨁◯ Moderate | IMPORTANT |
|  |  |  |  |  |  |  |  |  |  | **-- per 1 000** (from -- to --) |  |  |
|  |  |  |  |  |  |  |  |  |  | **-- per 1 000** (from -- to --) |  |  |

| **Certainty assessment** | | | | | | | **№ of patients** | | **Effect** | | **Certainty** | **Importance** |
| --- | --- | --- | --- | --- | --- | --- | --- | --- | --- | --- | --- | --- |
| **№ of studies** | **Study design** | **Risk of bias** | **Inconsistency** | **Indirectness** | **Imprecision** | **Other considerations** | **high PLR levels** | **low PLR levels** | **Relative (95% CI)** | **Absolute (95% CI)** |  |  |
| **PLR biomarker OS (assessed with: HR)** | | | | | | | | | | | | |
| 3 | observational studies | not serious | not serious | not serious | not serious | none |  |  | **HR 2.74** (1.74 to 4.31) [New outcome] | **-- per 1 000** (from -- to --) | ⨁⨁⨁◯ Moderate | IMPORTANT |
|  |  |  |  |  |  |  | - | 0.0% |  | **-- per 1 000** (from -- to --) |  |  |
| **PLR biomarker PFS (assessed with: HR)** | | | | | | | | | | | | |
| 3 | observational studies | not serious | not serious | not serious | not serious | none |  |  | **HR 2.25** (1.46 to 3.47) [New outcome] | **-- per 1 000** (from -- to --) | ⨁⨁⨁◯ Moderate | IMPORTANT |
|  |  |  |  |  |  |  | - | 0.0% |  | **-- per 1 000** (from -- to --) |  |  |

**Supplementary Figure 1.** Forrest plot representing pooled multivariate analysis of overall survival, NLR biomarker


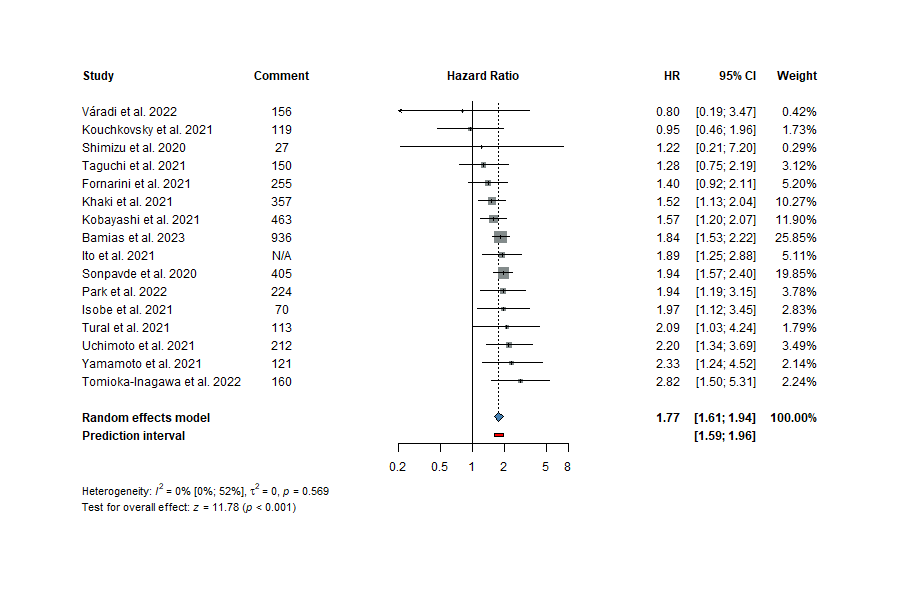


**Supplementary Figure 2.** Forrest plot representing pooled multivariate analysis of progression free survival, NLR biomarker


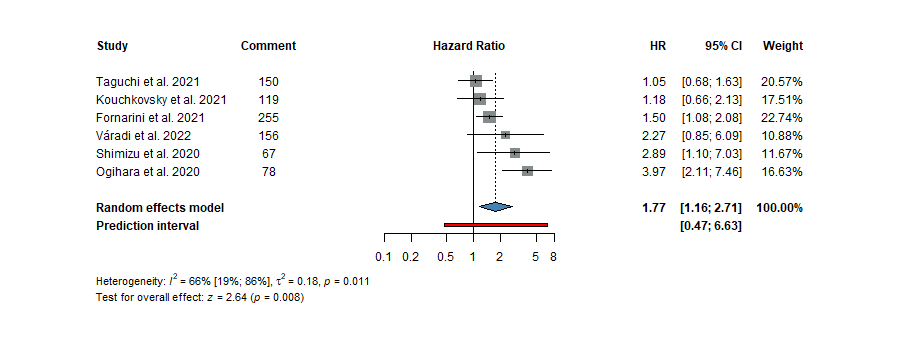


**Supplementary Figure 3.** Forrest plot representing objective response rates of NLR biomarker


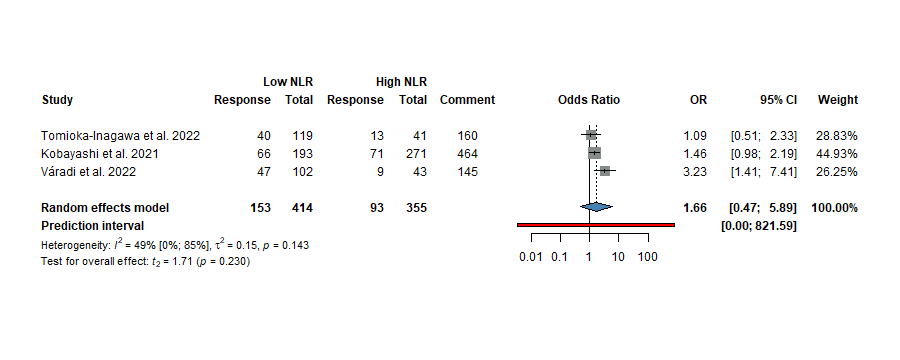


**Supplementary Figure 4.** Forrest plot representing pooled multivariate analysis of overall survival, CRP biomarker


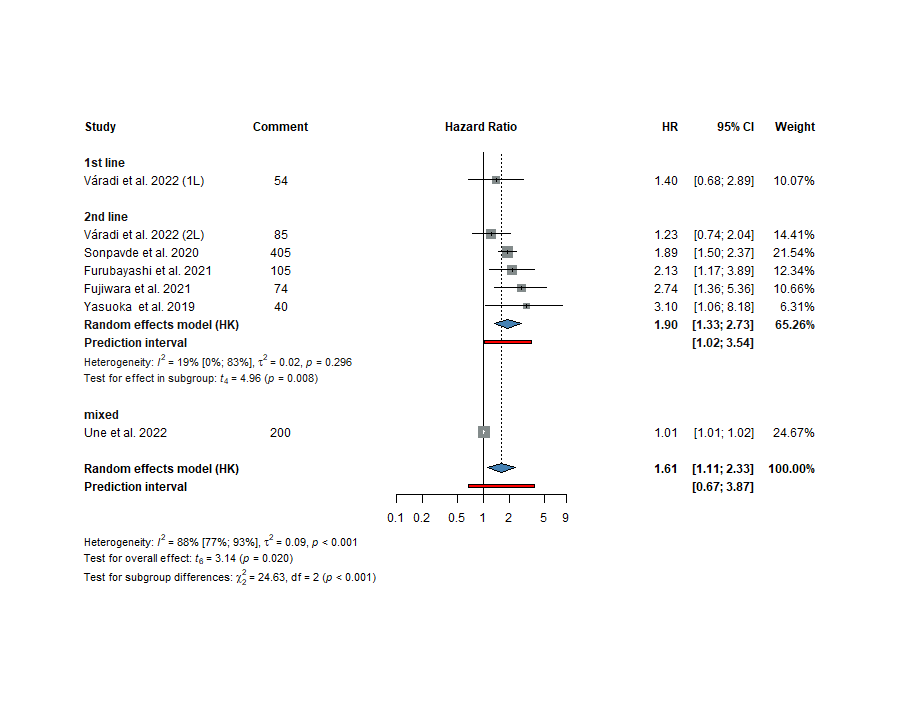


**Supplementary Figure 5.** Forrest plot representing subgroup analysis based on line of therapy pooled univariate analysis of overall survival, LDH biomarker


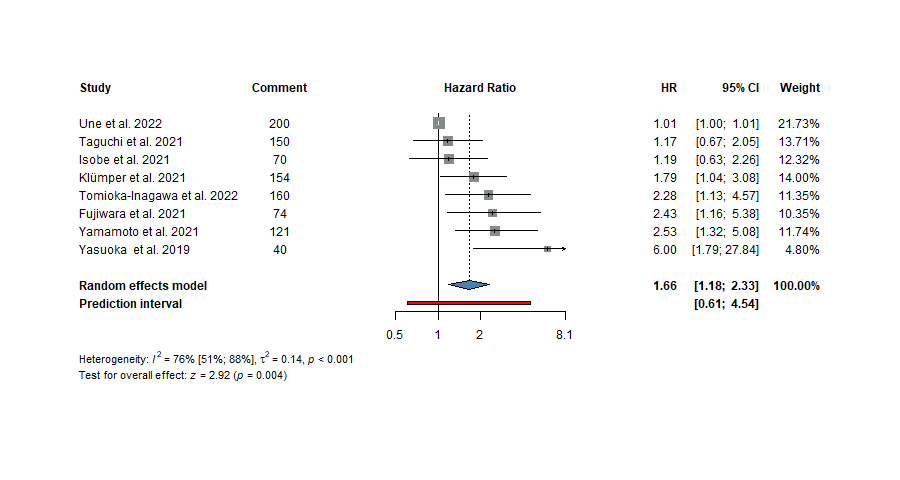


**Supplementary Figure 6.** Forrest plot representing pooled univariate analysis of overall survival, NLR biomarker


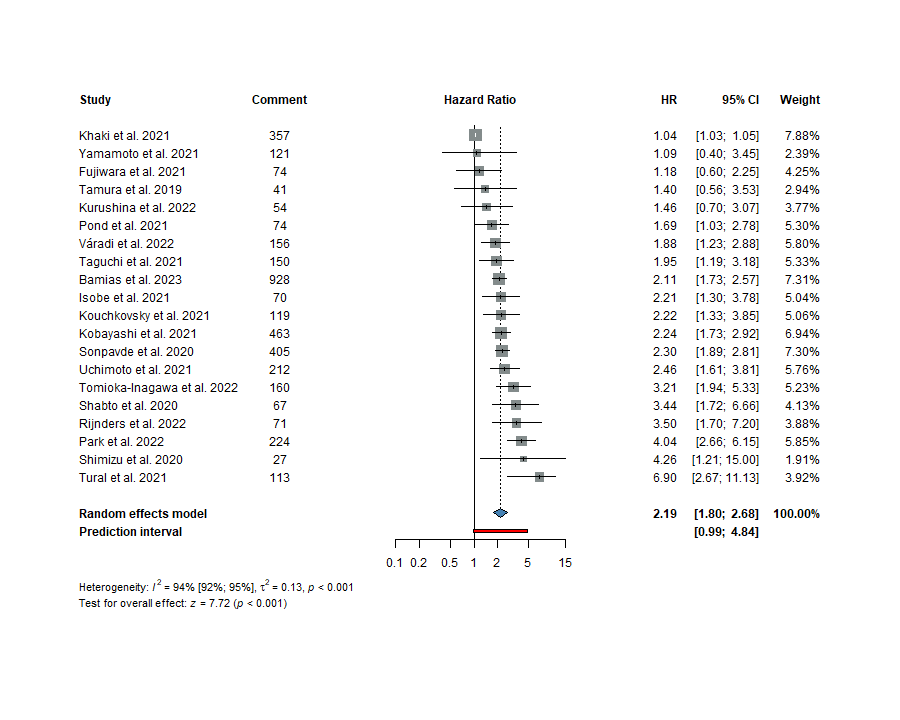


**Supplementary Figure 7.** Forrest plot representing pooled univariate analysis of progression free survival, NLR biomarker


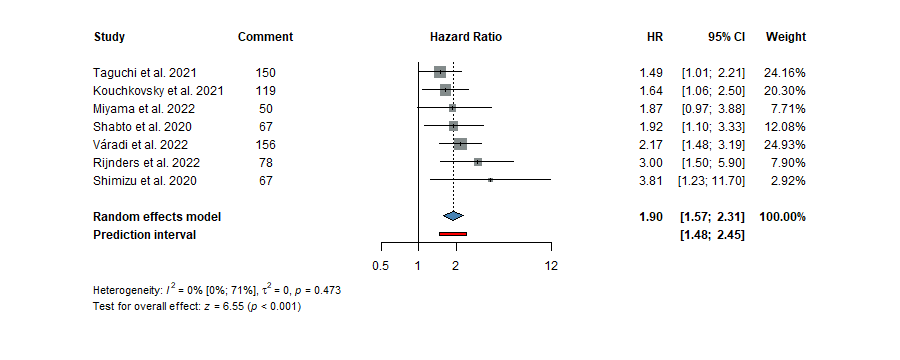


**Supplementary Figure 8.** Forrest plot representing subgroup analysis based on line of therapy pooled univariate analysis of overall survival, NLR biomarker


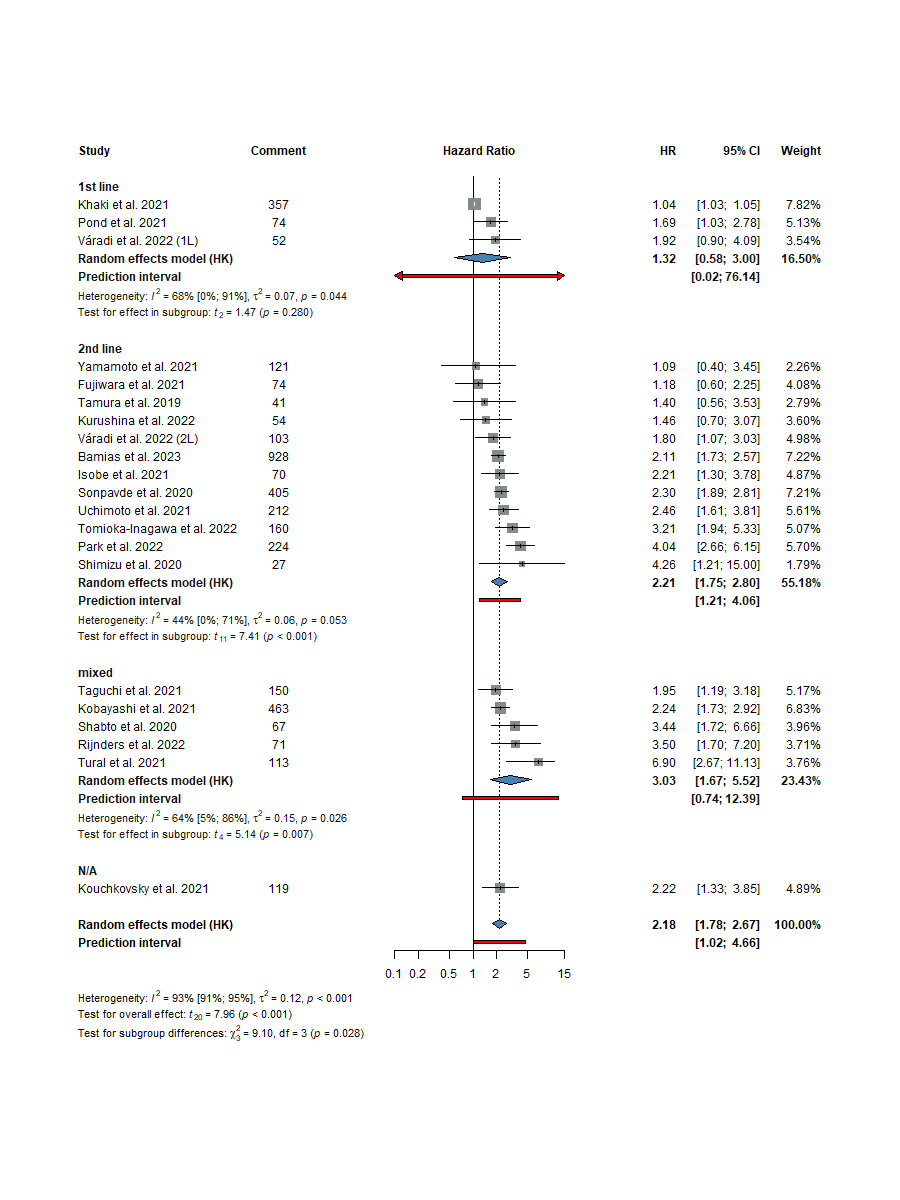


**Supplementary Figure 9.** Forrest plot representing subgroup analysis based on type of therapy pooled univariate analysis of overall survival, NLR biomarker


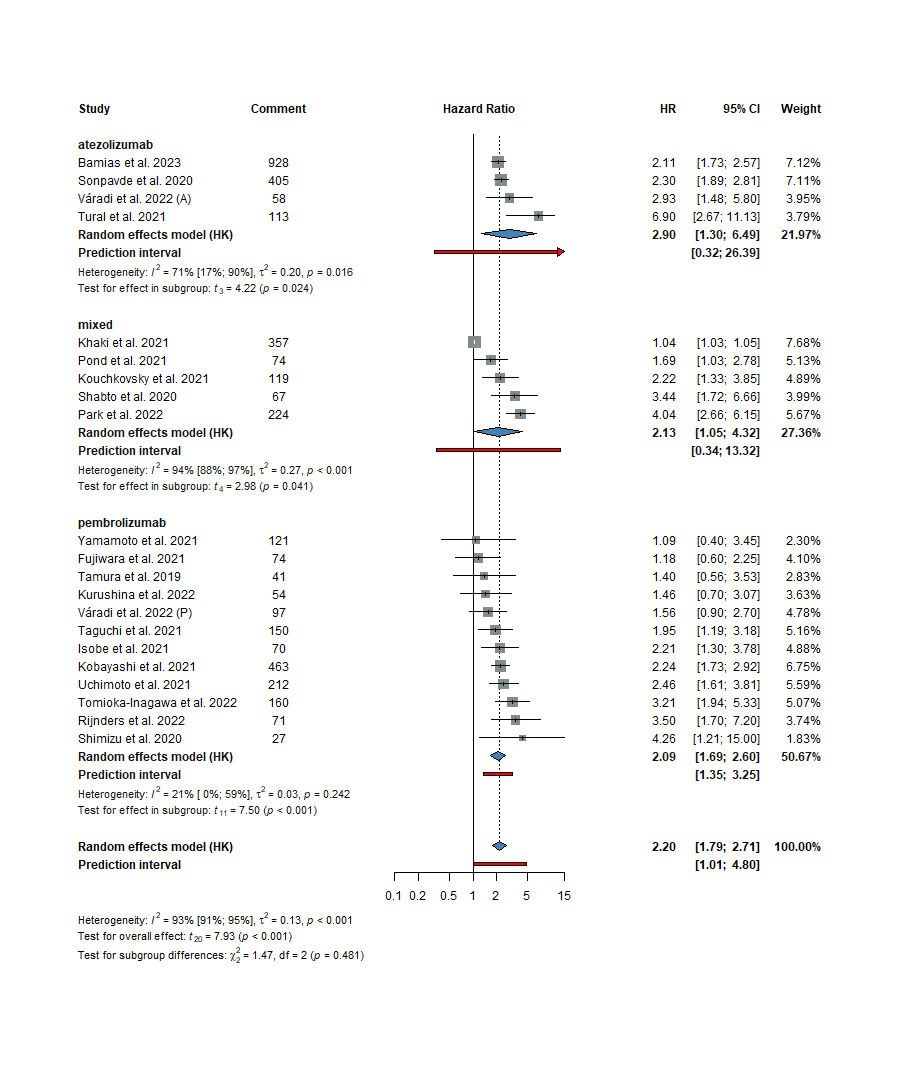


**Supplementary Figure 10.** Forrest plot representing subgroup analysis based on type of center pooled univariate analysis of overall survival, NLR biomarker


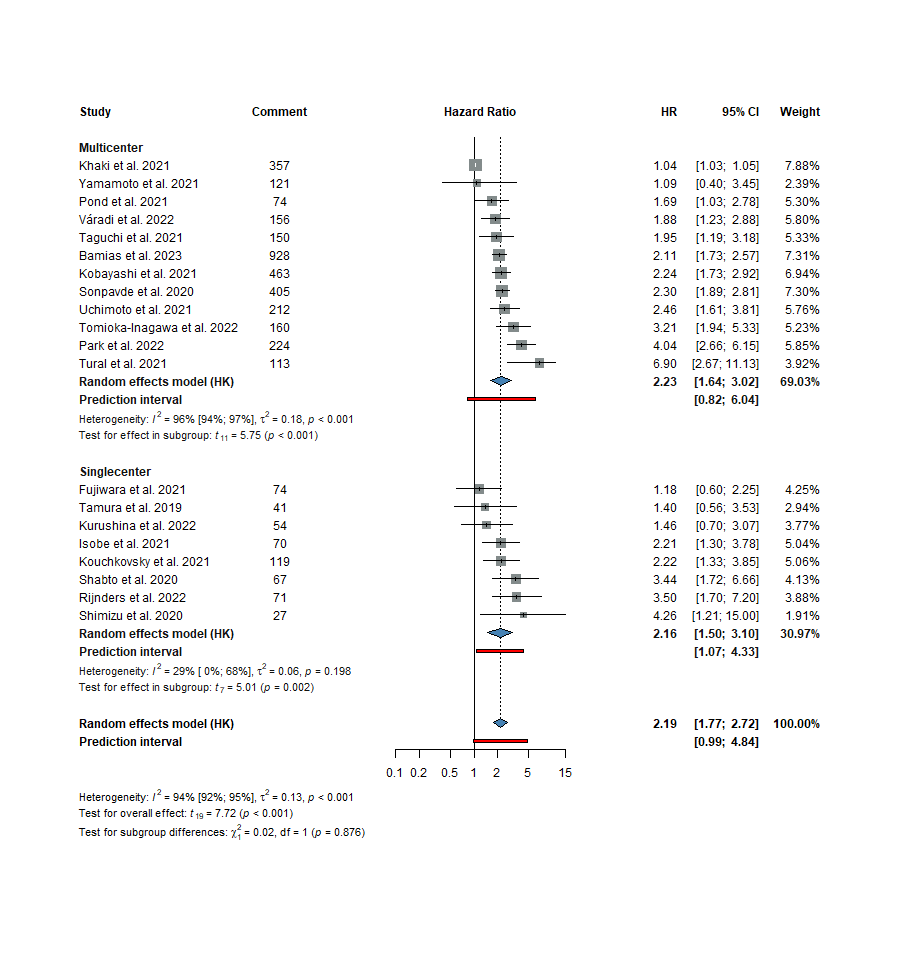


**Supplementary Figure 11.** Forrest plot representing subgroup analysis based on study design pooled univariate analysis of overall survival, NLR biomarker


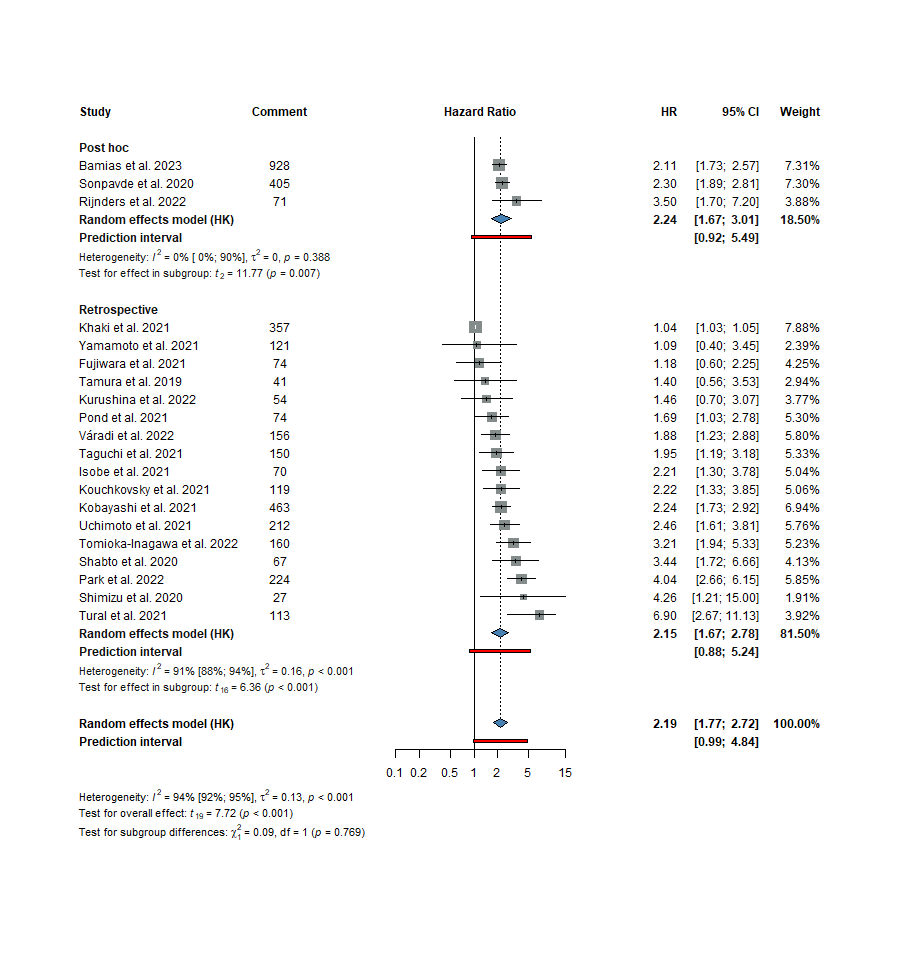


**Supplementary Figure 12.** Forrest plot representing subgroup analysis based on line of therapy pooled univariate analysis of progression free survival, NLR biomarker


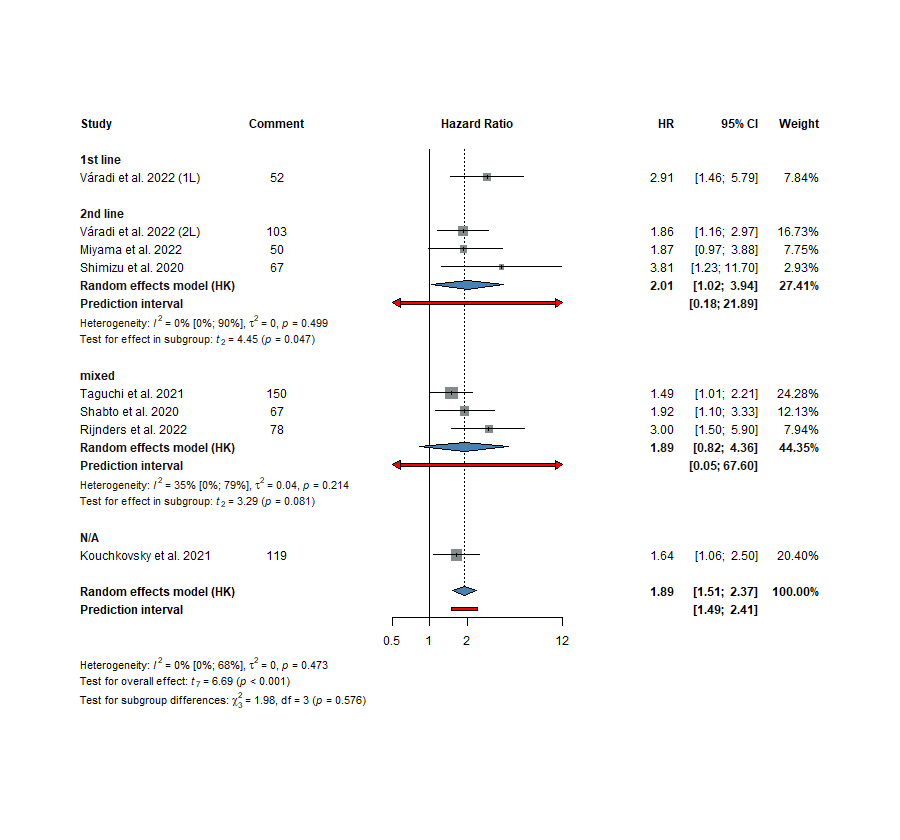


**Supplementary Figure 13.** Forrest plot representing subgroup analysis based on type of therapy pooled univariate analysis of progression free survival, NLR biomarker


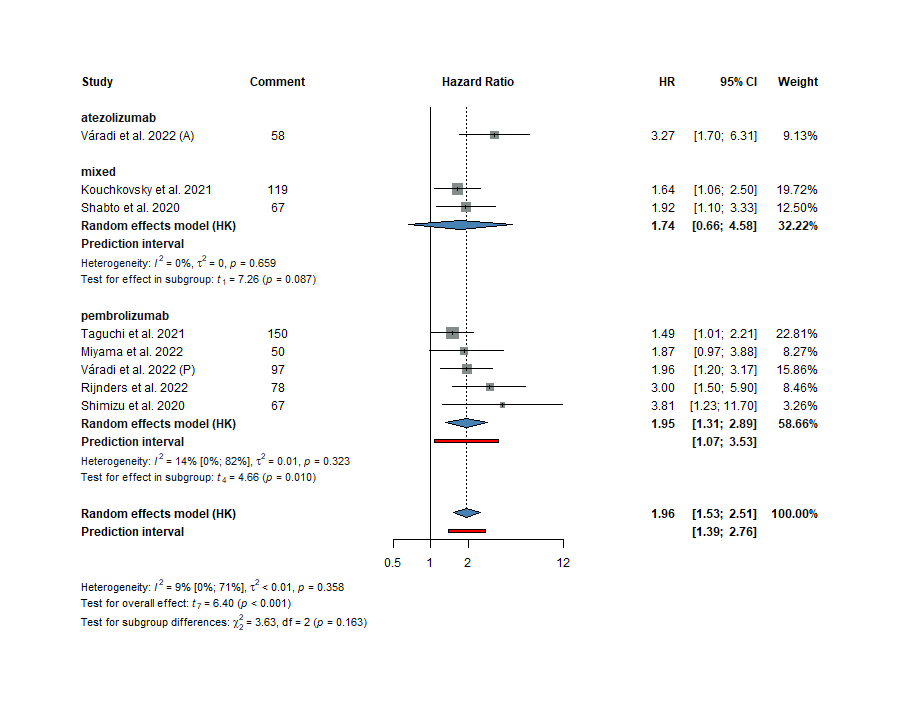


**Supplementary Figure 14.** Forrest plot representing subgroup analysis based on type of center pooled univariate analysis of progression free survival, NLR biomarker


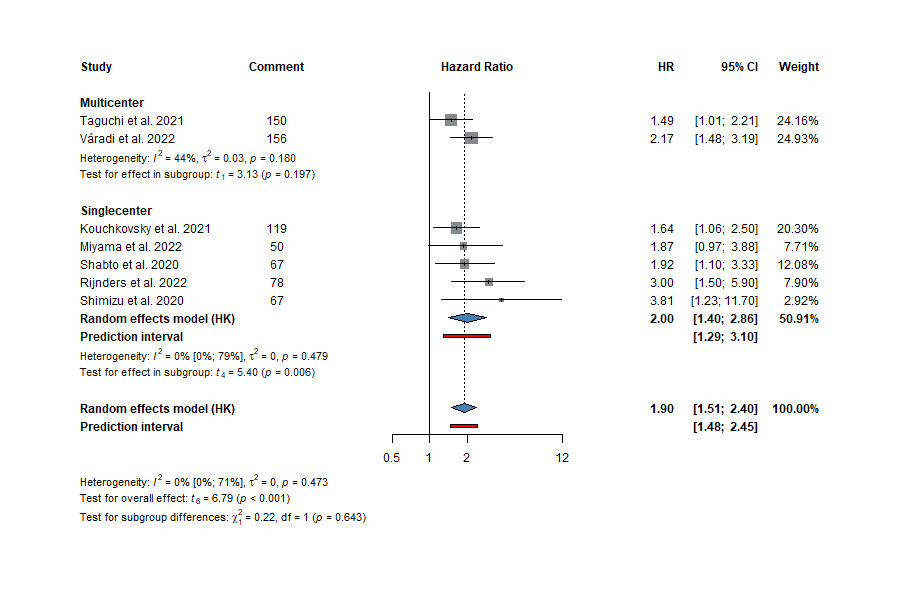


**Supplementary Figure 15.** Forrest plot representing subgroup analysis based on study design pooled univariate analysis of progression free survival, NLR biomarker
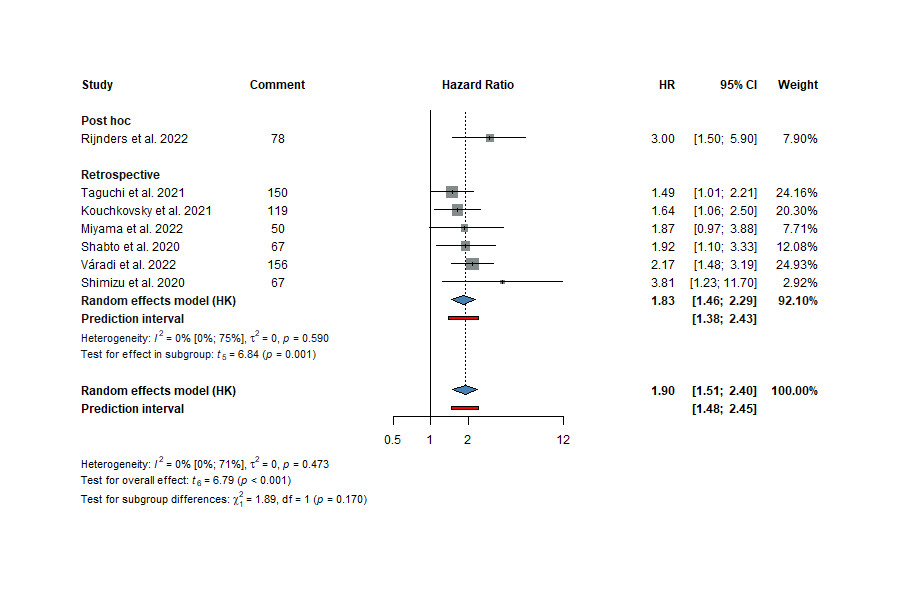


**Supplementary Figure 16.** Forrest plot representing pooled univariate analysis of overall survival, CRP biomarker


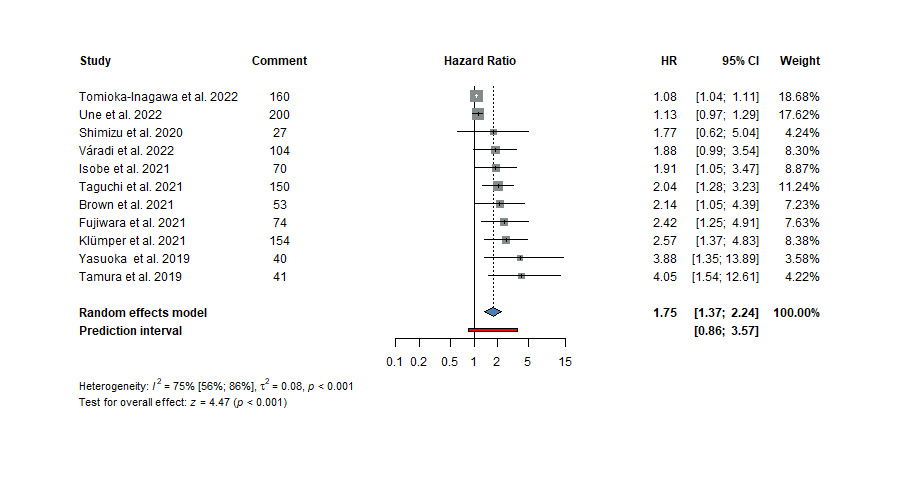


**Supplementary Figure 17.** Forrest plot representing pooled univariate analysis of progression free survival, CRP biomarker


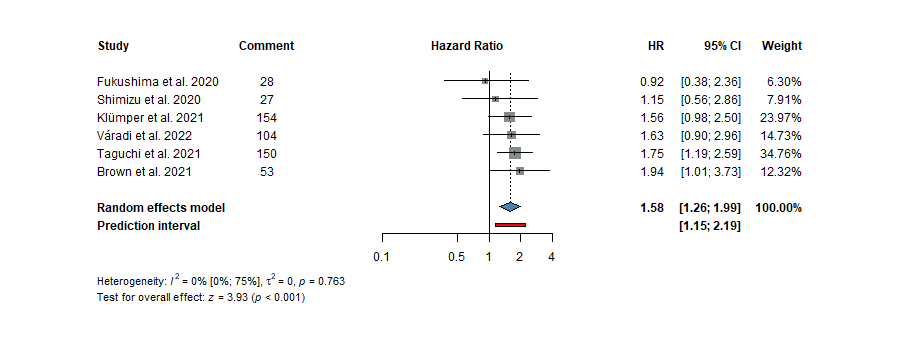


**Supplementary Figure 18.** Forrest plot representing subgroup analysis based on line of therapy pooled univariate analysis of overall survival, CRP biomarker


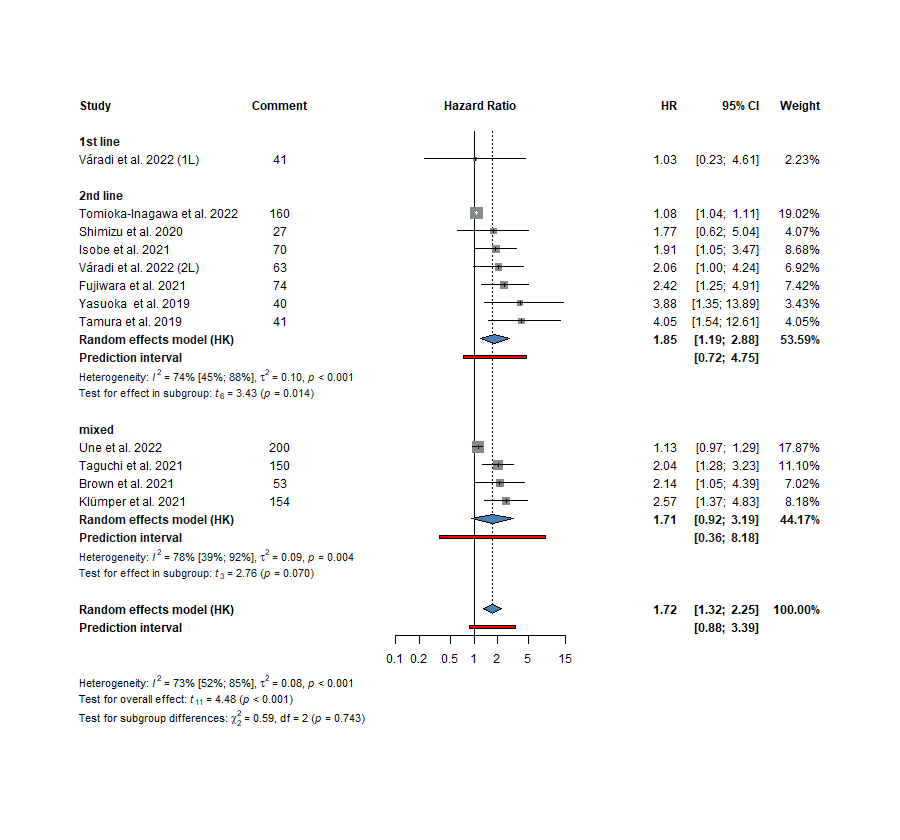


**Supplementary Figure 19.** Forrest plot representing subgroup analysis based on type of therapy pooled univariate analysis of overall survival, CRP biomarker


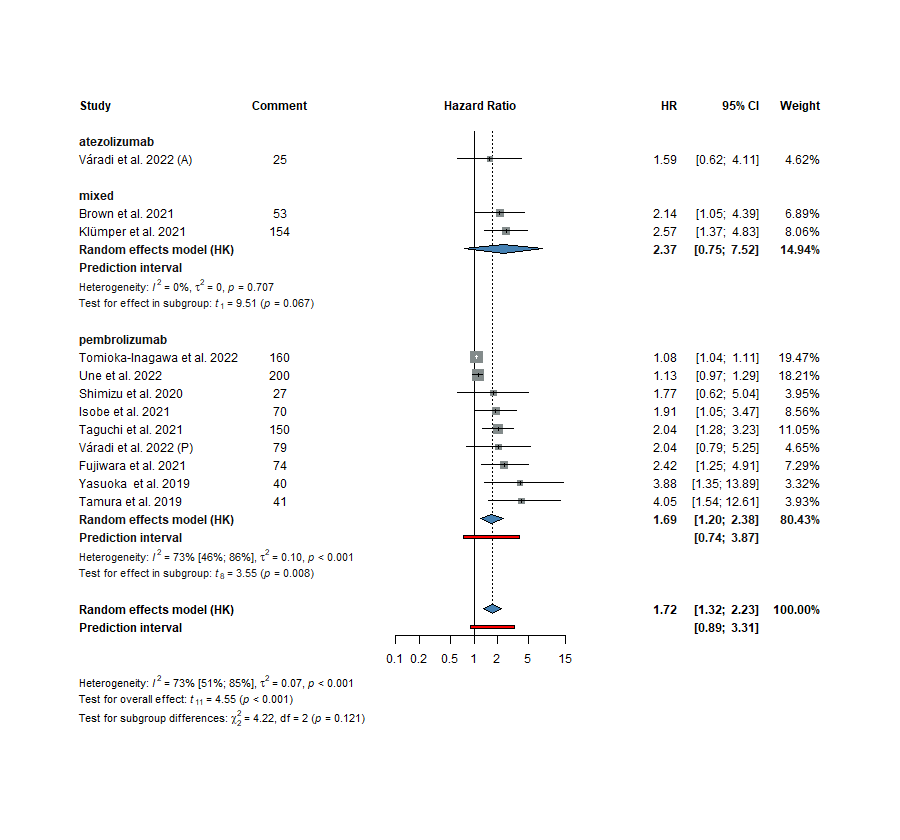


**Supplementary Figure 20.** Forrest plot representing subgroup analysis based on type of center pooled univariate analysis of overall survival, CRP biomarker


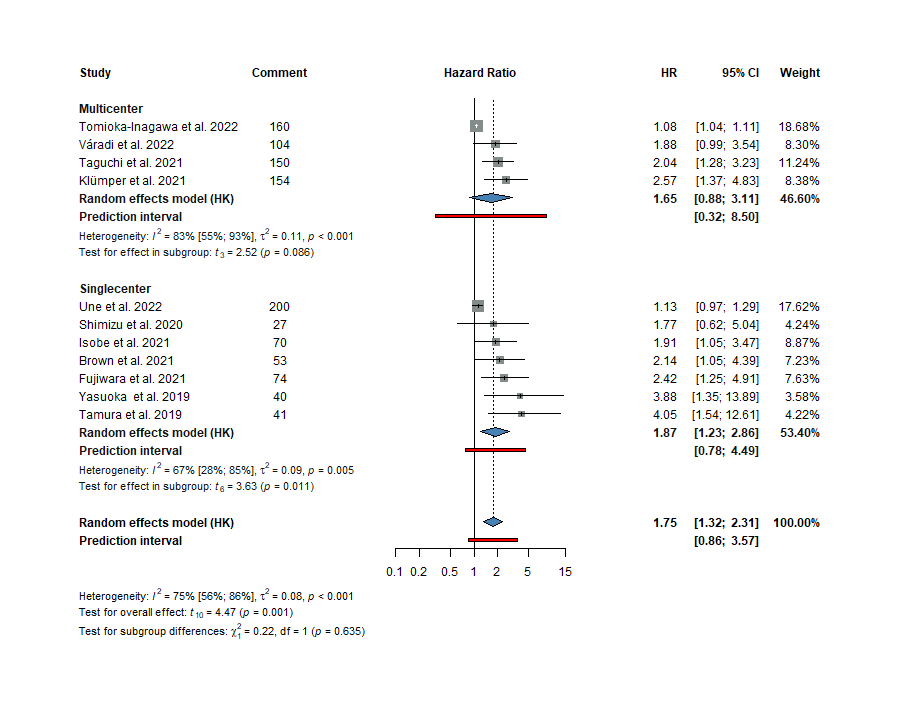


**Supplementary Figure 21.** Forrest plot representing subgroup analysis based on study design pooled univariate analysis of overall survival, CRP biomarker


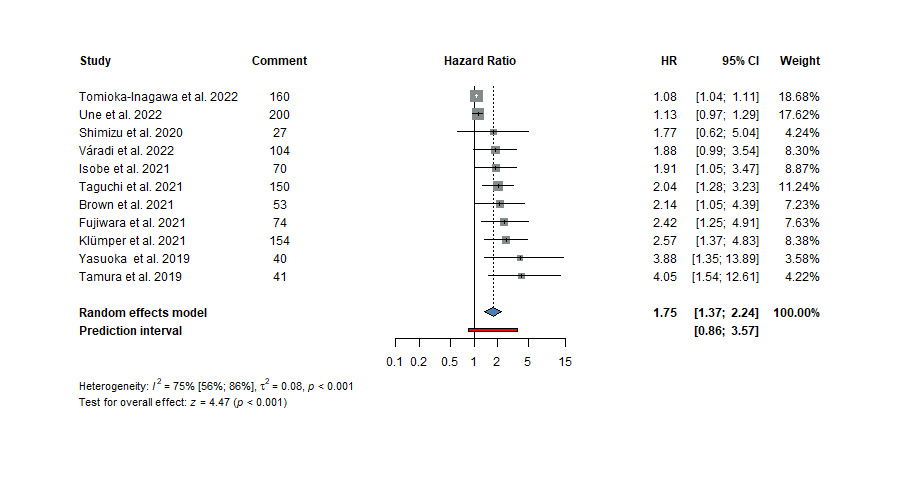


**Supplementary Figure 22.** Forrest plot representing subgroup analysis based on line of therapy pooled univariate analysis of progression free survival, CRP biomarker

**
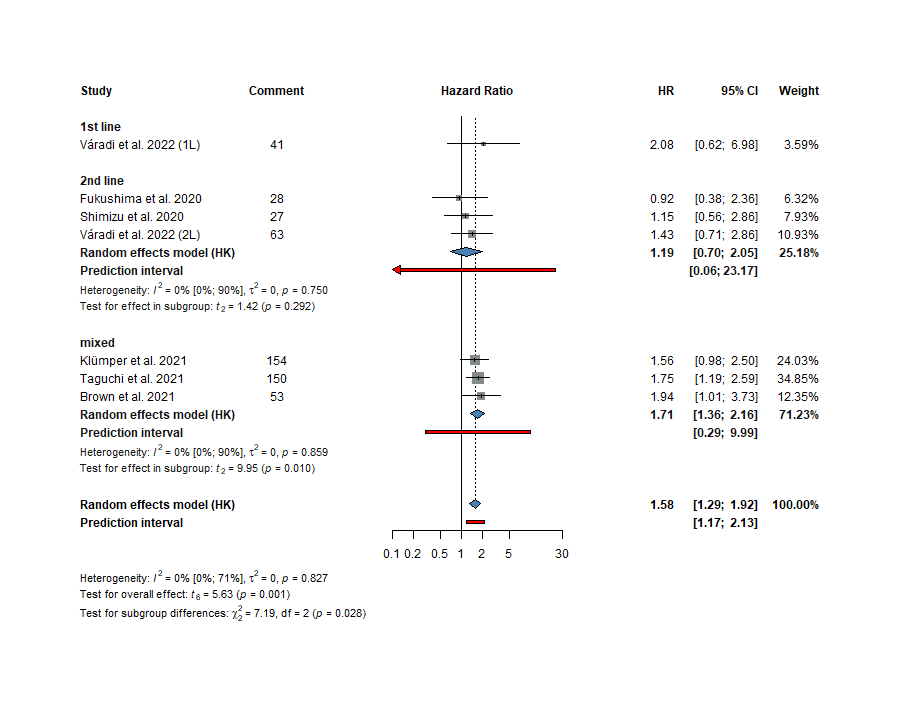
**

**Supplementary Figure 23.** Forrest plot representing subgroup analysis based on type of therapy pooled univariate analysis of progression free survival, CRP biomarker


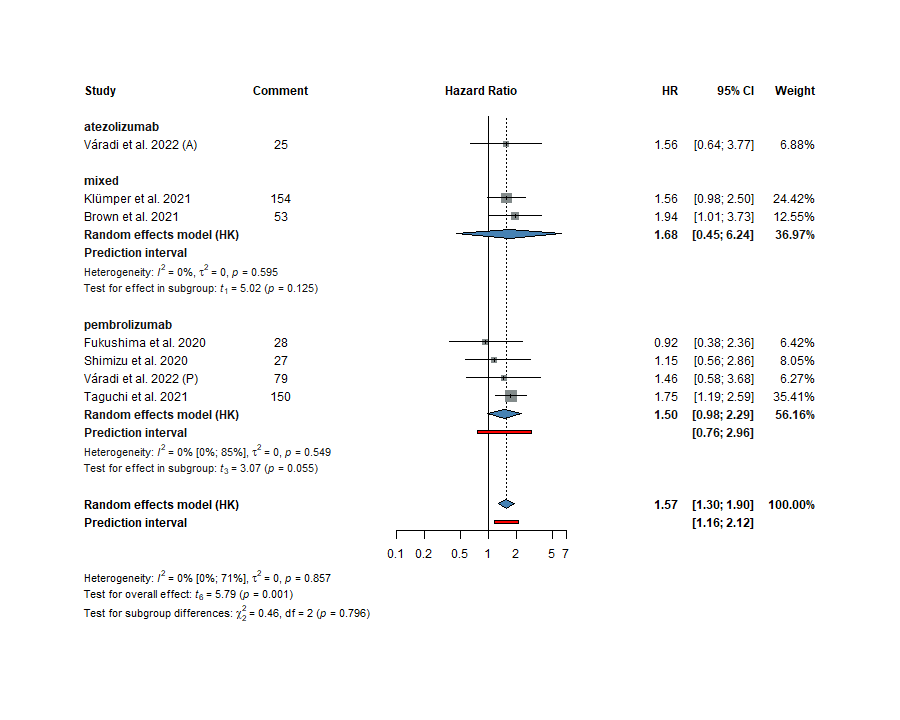


**Supplementary Figure 24.** Forrest plot representing subgroup analysis based on type of center pooled univariate analysis of progression free survival, CRP biomarker


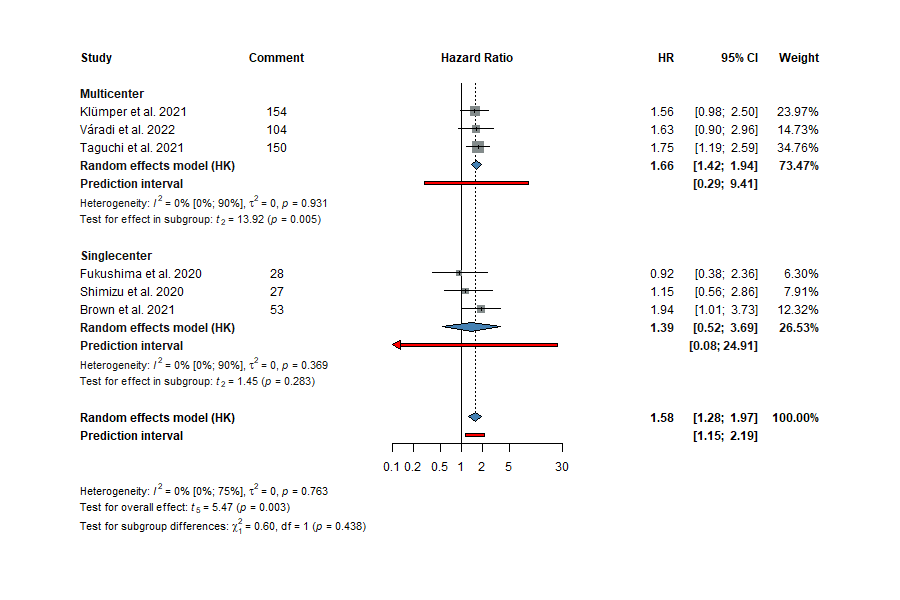


**Supplementary Figure 25.** Forrest plot representing subgroup analysis based on study design pooled univariate analysis of progression free survival, CRP biomarker


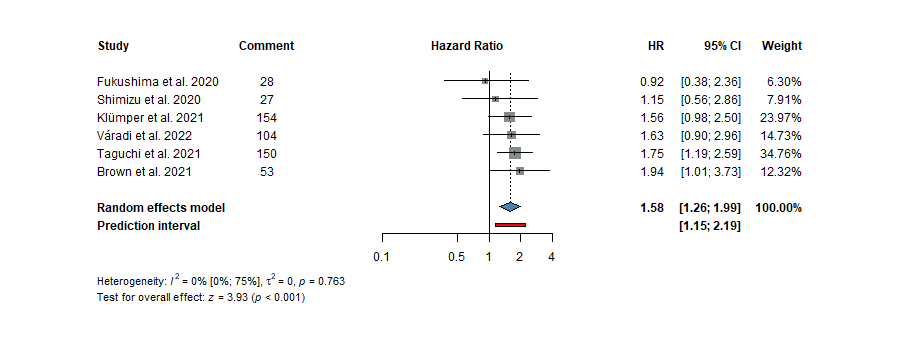


**Supplementary text 1.**

(urothelial or "urothelial cancer" or "bladder cancer") AND ("immunecheckpoint inhibitor" or nivolumab or durvalumab or atezolizumab or avelumab or pembrolizumab) AND (NLR or "neutrophil-to-lymphocyte ratio" or PLR or "platelet-to-lymphocyte ratio" or CRP or "C reactive protein" or LDH or "lactatedehydrogenase")
